# Gut–Metabolome–Proteome Interactions in Age‐Related Hearing Loss: Insights from Fecal Microbiota Transplantation and Multi‐Omics Analyses

**DOI:** 10.1002/advs.202514269

**Published:** 2026-01-31

**Authors:** Ting Yang, Ziwen Gao, Hui Huang, Chanyuan Zhang, Yiquan Tang, Qianhui Qu, Huabin Li, Jing Ke, Zhiji Chen, Menglong Feng, Hu Zhou, Yilai Shu, Wei Yuan

**Affiliations:** ^1^ Department of Otorhinolaryngology Head and Neck Surgery Chongqing General Hospital Chongqing University Chongqing China; ^2^ ENT Institute and Department of Otorhinolaryngology Eye and ENT Hospital Fudan University Shanghai China; ^3^ Institutes of Biomedical Science Fudan University Shanghai China; ^4^ NHC Key Laboratory of Hearing Medicine Shanghai China; ^5^ State Key Laboratory of Medical Neurobiology and MOE Frontiers Center for Brain Science Fudan University Shanghai China; ^6^ School of Pharmaceutical Science and Technology Hangzhou Institute for Advanced Study University of Chinese Academy of Sciences Hangzhou China; ^7^ Department of Analytical Chemistry State Key Laboratory of Drug Research Shanghai Institute of Materia Medica Chinese Academy of Sciences Shanghai China; ^8^ Institutes of Biomedical Sciences Shanghai Key Laboratory of Medical Epigenetics International Co‐laboratory of Medical Epigenetics and Metabolism (Ministry of Science and Technology) Department of Systems Biology for Medicine Fudan University Shanghai China

**Keywords:** 5‐hydroxytryptophan, age‐related hearing loss, fecal microbiota transplantation, germ‐free mice, gut microbiota, multi‐omics integration

## Abstract

Age‐related hearing loss (ARHL) is a prevalent sensory disorder lacking disease‐modifying interventions. The biological drivers, particularly the contribution of the gut microbiota and gut–inner ear crosstalk, remain poorly defined. Here, we utilize germ‐free (GF) mice and fecal microbiota transplantation (FMT) to isolate microbiota‐dependent effects on ARHL progression. Through integrated metagenomic, metabolomic, and proteomic profiling, we map molecular signatures of auditory aging and uncover functional gut‐inner ear network, prioritizing 5‐hydroxytryptophan (5‐HTP) as a key intermediate metabolite within this network. Furthermore, in an aging‐like House Ear Institute–Organ of Corti 1 (HEI‐OC1) model, 5‐HTP exhibits protective effects, potentially mediated through the PI3K/Akt–antioxidant signaling axis. Collectively, this study provides a valuable multi‐omics resource and highlights microbiota‐derived metabolic regulation as a promising avenue for biomarker discovery and therapeutic development in ARHL.

## Graphical abstract

Germ‐free (GF) mice receiving fecal microbiota transplantation (FMT) reveal microbiota‐dependent effects on auditory aging. Integrated metagenomic, metabolomic and proteomic profiling maps gut–inner ear network and highlights 5‐hydroxytryptophan (5‐HTP) as a microbiota‐linked metabolic hub in age‐related hearing loss (ARHL). Cellular validation shows that 5‐HTP attenuates aging‐like stress through the PI3K/Akt–antioxidant signaling axis, supporting microbiome‐based diagnostic and therapeutic strategies as promising avenues for ARHL.

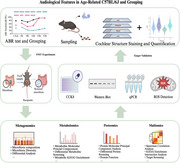

## 1 Introduction

Age‐related hearing loss (ARHL), or presbycusis, is a symmetrical, progressive, age‐dependent decline in auditory sensitivity that predominantly affects high frequencies and arises from sensorineural pathology [1]. Demographic projections indicate that the global population aged 60 years and older will reach approximately 2.1 billion by 2050 [2]. Epidemiological evidence has demonstrated a marked increase in the risk of hearing impairment beyond the age of 40, with more than one‐third of individuals aged 65 and older anticipated to experience disabling hearing loss [3]. Despite extensive research efforts, the precise pathophysiological mechanisms underlying ARHL remain poorly defined [4], and no disease‐modifying pharmacological interventions are currently available. Clinical management relies exclusively on rehabilitative strategies, including hearing aids, cochlear implants, and auditory training, which aim to enhance speech perception, communication abilities, and overall quality of life [5]. ARHL thus represents a substantial and growing public health challenge, imposing significant detrimental effects on individual well‐being and a considerable socioeconomic burden.

In recent years, researchers have attempted to understand the potential pathomechanisms of ARHL via omics approaches [6, 7, 8, 9]; however, definitive biomarkers are still lacking. Furthermore, accumulating evidence implicates the gut microbiota in neuroinflammatory processes: dysbiosis can increase the production of proinflammatory cytokines and intestinal permeability, promoting systemic inflammation that compromises blood–brain barrier (BBB) integrity [10, 11]. Analogous to the BBB, the blood–labyrinth barrier (BLB) limits the entry of toxins, pathogens, and inflammatory mediators into the inner ear [12, 13].

Emerging data indicate that intestinal inflammation can exacerbate hearing loss, whereas modulating the gut microbial composition can mitigate noise‐induced hearing loss (NIHL) by dampening cochlear immune and inflammatory responses [14, 15]. These observations seem to suggest existence of a gut–inner ear axis; however, the relationship between ARHL and the gut microbiota, and the mechanisms linking microbial dysbiosis to auditory senescence, remain poorly defined. Identifying key molecular mediators along this axis may reveal novel strategies for prevention or intervention.

**FIGURE 1:**
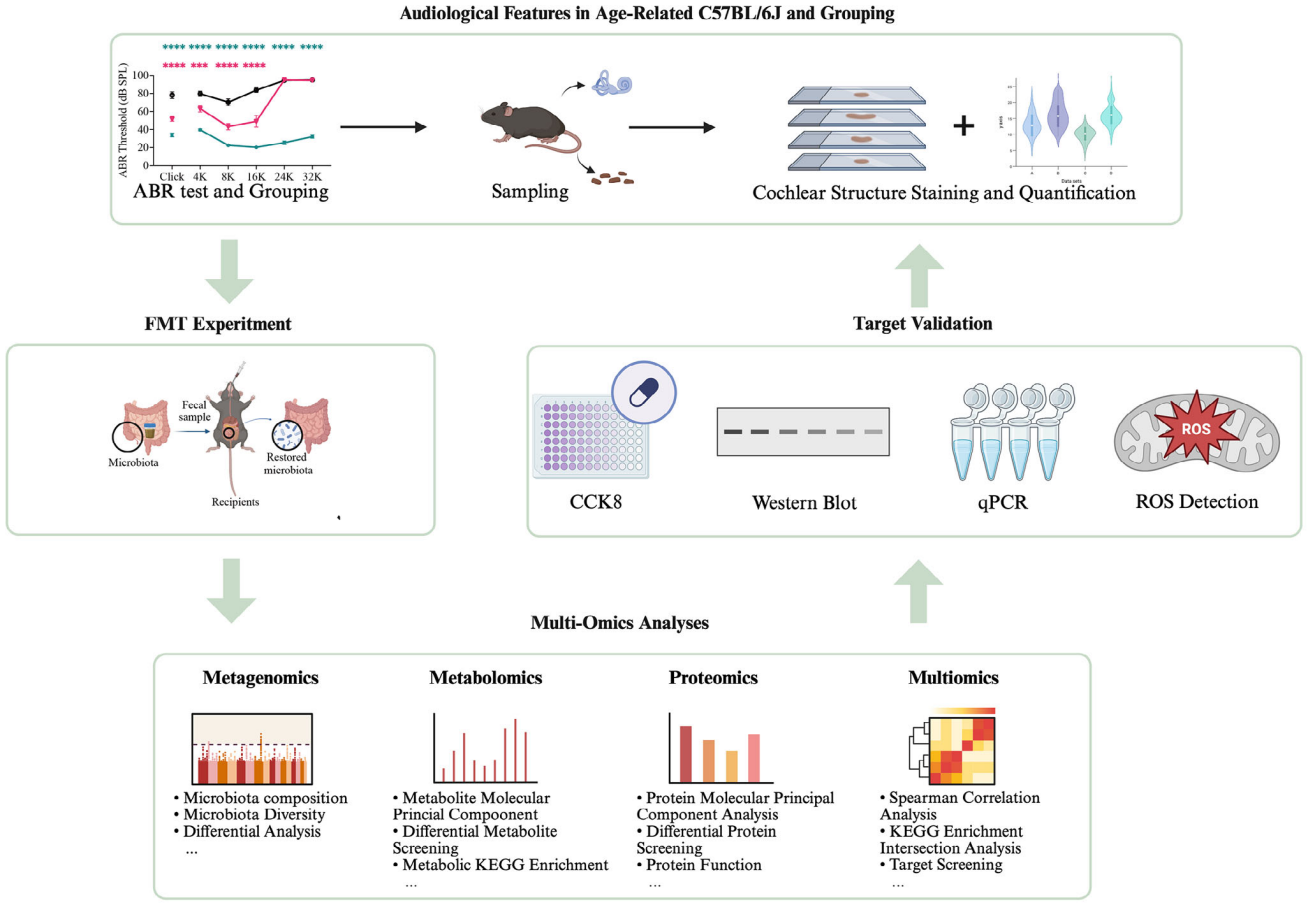
Illustration of the study design. Figure created with BioRender (https://biorender.com/).

Here, we combined germ‐free (GF) mouse models with fecal microbiota transplantation (FMT) to assess the direct effects of donor microbiota on ARHL progression. We then performed integrated metagenomic, metabolomic, and proteomic profiling to define ARHL‐associated signatures and cross‐level interactions. This multi‐omics framework identified 5‐hydroxytryptophan (5‐HTP) as a candidate gut–inner ear mediator. In vitro, 5‐HTP conferred protective effects in a House Ear Institute‐Organ of Corti (HEI‐OC1) model of aging (Figure 1).

## 2 Results

### 2.1 Audiological Features of Changes in the Cochleae of Aging C57BL/6J Mice

**FIGURE 2:**
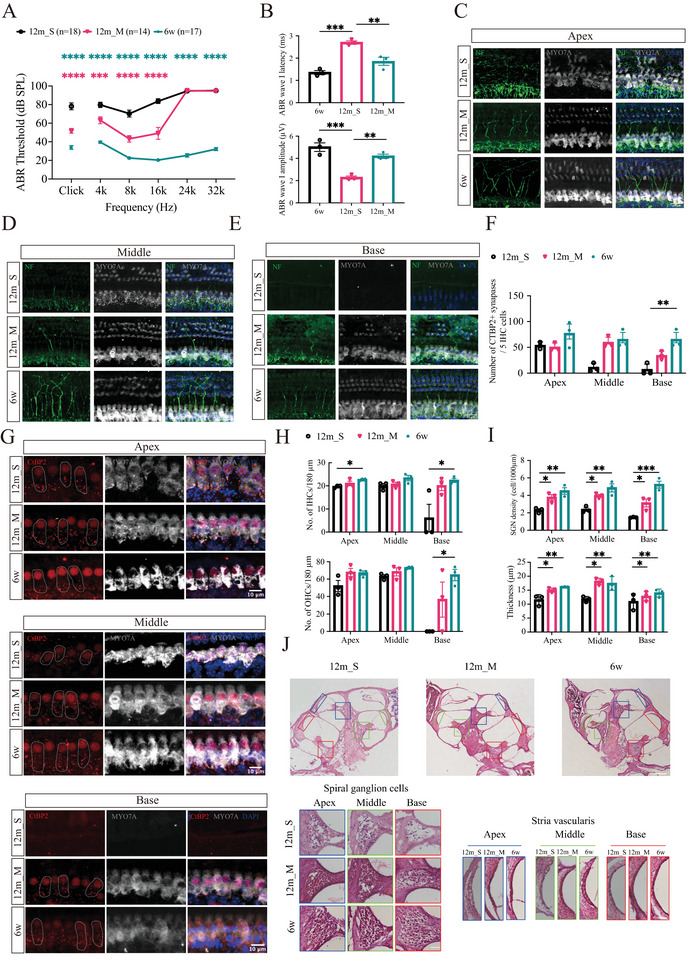
Hearing thresholds and evaluation of cochlear structure in different groups. (A) ABR thresholds at click and at 4, 8, 16 24, and 32kHz were measured for each group. (*n* = 18 for 12m_S, *n* = 14 for 12m_M, and *n* = 17 for 6w). ***p < 0.001, ****p < 0.0001 (pink asterisks indicate 12m_M vs. 12m_S; green asterisks indicate 6w vs. 12m_S). One‐way ANOVA test. (B) ABR wave I amplitudes and latencies at 8kHz. **p < 0.01 and ***p < 0.001. One‐way ANOVA. (C–E) Confocal images of HCs (MYO7A, gray), ANFs (NF, green), and nuclei (DAPI, blue). Scale bar, 20µm. (F) Statistical analysis of the data in (G) (*n* = 3). **p < 0.01. One‐way ANOVA. (G) Confocal images of HCs (MYO7A, gray), presynaptic ribbons (CtBP2, red), and nuclei (DAPI, blue). The area outlined by the white dashed line highlights the synaptic dots located in IHC. Scale bar, 10µm. (H) Statistical analysis of the data in (C–E) (*n* = 3). *p<0.05. One‐way ANOVA. I) Statistical analysis of (J) (*n* = 3). *p < 0.05, **p < 0.01, ***p < 0.001. One‐way ANOVA test. (J) Image of H&E‐stained cochleae from each group showing the SGNs and SV at the apex (blue), middle (green), and base (red). Scale bar, 100/50 µm.

Given the similarities in the auditory systems of mice and humans, coupled with the short lifespan and genetic standardization of mice, mouse models have proven invaluable for studying the cellular and genetic bases of ARHL. Among mouse strains, the C57BL/6J strain is the most widely used in research. Mice of this inbred strain exhibit precocious and progressive hearing loss, with extensive degeneration observed in various components of the cochlea and auditory cortex, which aligns closely with the major types of ARHL [16, 17, 18, 19]. Here, hearing thresholds at various frequencies were assessed by measuring the auditory brainstem response (ABR) in C57BL/6J mice. At 6 weeks (6w) of age, the hearing of the mice was normal, however, the hearing threshold significantly increased in the 12‐month‐old (12m) group, particularly at high frequencies. Notably, substantial variability in hearing thresholds was observed among individual mice within the 12m group (Figure 2A).

Although ABR thresholds reflect hair cell (HC) function, they are relatively insensitive to neural damage in the cochlea. We therefore analyzed ABR wave I amplitude and latency as indices of auditory nerve fiber (ANF) integrity [20]. Because aged mice have elevated thresholds, suprathreshold wave I responses were recorded at 8kHz. Our results demonstrated that the wave I amplitude in the 6w and 12m mice with moderate hearing loss (12m_M) groups was significantly greater than that observed in the 12m mice with severe hearing loss (12m_S) group. Furthermore, the wave I latency in the 12m_S group was significantly prolonged compared with both the 6w and 12m_M groups (Figure 2B). This result was likely due to a reduction in synchronous firing, lower discharge rates, and decreased recruitment of ANFs. Finally, we classified and clustered these mice into three groups based on the severity of hearing loss: 6w, 12m_M and 12m_S [21] (Figure 2A).

Hematoxylin and eosin (H&E) and histological immunofluorescence staining were performed to evaluate the cochlear structures. As shown in Figure 2, the main anatomical structures in the auditory pathway exhibited various degrees of morphological damage in the 12m groups. The mice in the 12m groups presented varying degrees of hearing loss (HL) and damage to auditory structures, including inner hair cells (IHCs), outer hair cells (OHCs), presynaptic ribbons, and ANFs, particularly at the base turn of the cochlea (Figure 2C–H). Although some results obtained from the staining and quantification of cochlear structures did not reach statistical significance, a general trend of greater structural integrity in 6w mice was noted. Moreover, compared with the 12m_S group, the 12m_M group exhibited better preservation of cochlear structures. Notably, the most significant differences among the groups were observed in the reduction in the number of spiral ganglion neurons (SGNs) and stria vascularis (SV) (Figure 2I,J). Overall, aging in C57BL/6J mice is associated with progressive auditory dysfunction and structural degeneration, most severe in the 12m_S group.

### 2.2 FMT Intervention Ameliorated ARHL in GF Mice

**FIGURE 3:**
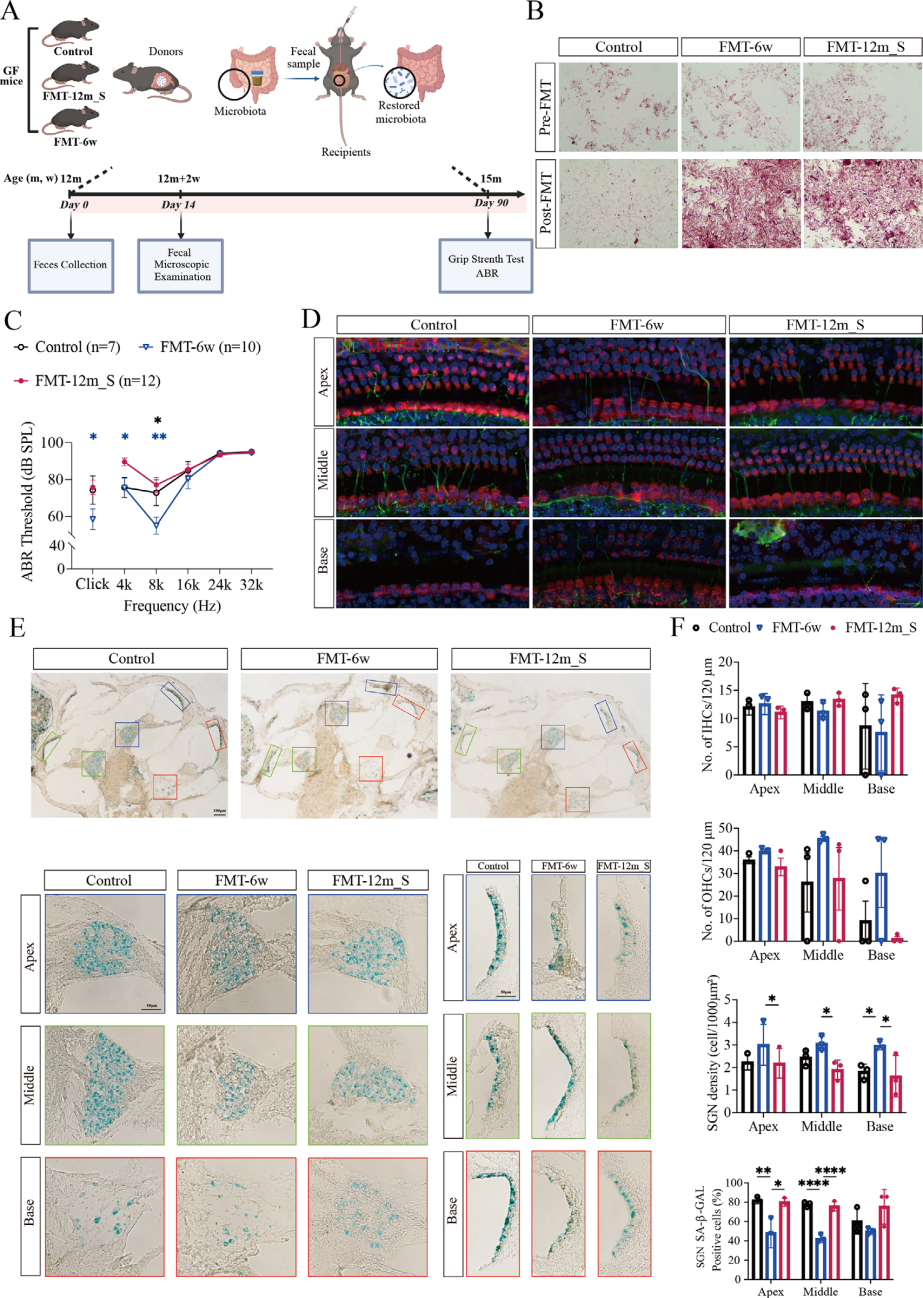
Efficacy evaluation of FMT in GF mice. (A) FMT intervention protocol. The figure was created with BioRender (https://biorender.com/). (B) Gram staining and microscopic examination of GF mouse fecal samples before and after FMT. Scale bar, 10µm. (C) ABR thresholds at clickand at 4, 8, 16, 24; and 32kHz were measured for each group (*n* = 7 for the control group; *n* = 10 for the FMT‐6w group, and *n* = 12 for the FMT‐12m_S group). *p < 0.05 and **p < 0.01 (blue asterisks indicate FMT‐6w vs. FMT‐12m_S; black asterisks indicate FMT‐6w vs. Control). One‐way ANOVA. (D) Confocal images of HCs (MYO7A, red), ANFs (NF, green), and nuclei (DAPI, blue) in each group. Scale bar, 20µm. (E) SA‐β‐GAL staining of cochlear tissues from different groups. Apex (blue), middle (green), and base (red). Scale bar, 100/50µm. (F) Statistical analysis of the data in (D‐E, S1A) (*n* = 3). *p < 0.05, **p < 0.01, ****p < 0.0001. One‐way ANOVA test.

To probe the causal role of the gut microbiota in ARHL progression, we performed FMT in a GF mouse model (Figure 3A). Fecal samples from 6w and 12m_S donors were transplanted into 12m GF recipients. Gram staining and microscopic examinations confirmed engraftment: no bacteria were detected before FMT, whereas robust colonization was evident two weeks post‐FMT (Figure 3B). Auditory testing revealed striking intergroup differences (Figure 3C). FMT‐6w recipients exhibited significantly lower ABR thresholds (better hearing) than both untreated GF controls and FMT‐12m_S recipients did. Analysis of ABR waveforms showed that the shortest wave I latencies in FMT‐6w mice, although this difference did not reach statistical significance (Figure S1D). Notably, FMT‐12m_S recipients displayed significantly reduced wave I amplitudes compared with FMT‐6w mice, with the control group showing intermediate values (Figure S1D).

Histological analysis of the cochlea supported these functional findings. Compared with the FMT‐12m_S and GF control groups, the FMT‐6w group tended to show better preservation of IHCs and OHCs in most cochlear turns, although these differences were not statistically significant; additionally, the ANF density appeared greater (Figure 3D,F). No significant differences in SV morphology were detected among the groups (Figure S1B). Notably, compared with organ of Corti, SGNs displayed greater responsiveness to FMT, which is consistent with the heightened aging susceptibility of SGNs described in Section 2.1 (Figure 2H,I, Figure 3f, Figure S1A). To assess cellular senescence, senescence‐associated β‐galactosidase (SA‐β‐gal) staining was performed on cochlear sections. Positive signals localized primarily in the SV and SGN regions. Compared with the FMT‐12m_S and GF control groups, the FMT‐6w group displayed fewer SA‐β‐GAL‐positive SGNs, suggesting that FMT from young donors may attenuate cellular senescence in key cochlear structures (Figure 3E,F).

In addition, forelimb grip strength was significantly lower in the GF controls than in the FMT recipients (Figure S1E). Furthermore, the grip strength of FMT‐6w mice was significantly greater than that of FMT‐12m_S and control mice. We also noted that intestinal permeability in FMT‐12m_S mice was higher than in the other two groups, although the pairwise differences did not reach statistical significance (Figure S1C). Collectively, these findings indicate that, in GF mice, FMT from different donor groups differentially shapes ARHL‐related phenotypes and systemic performance.

### 2.3 Alterations in the Gut Microbiota Composition in ARHL

**FIGURE 4:**
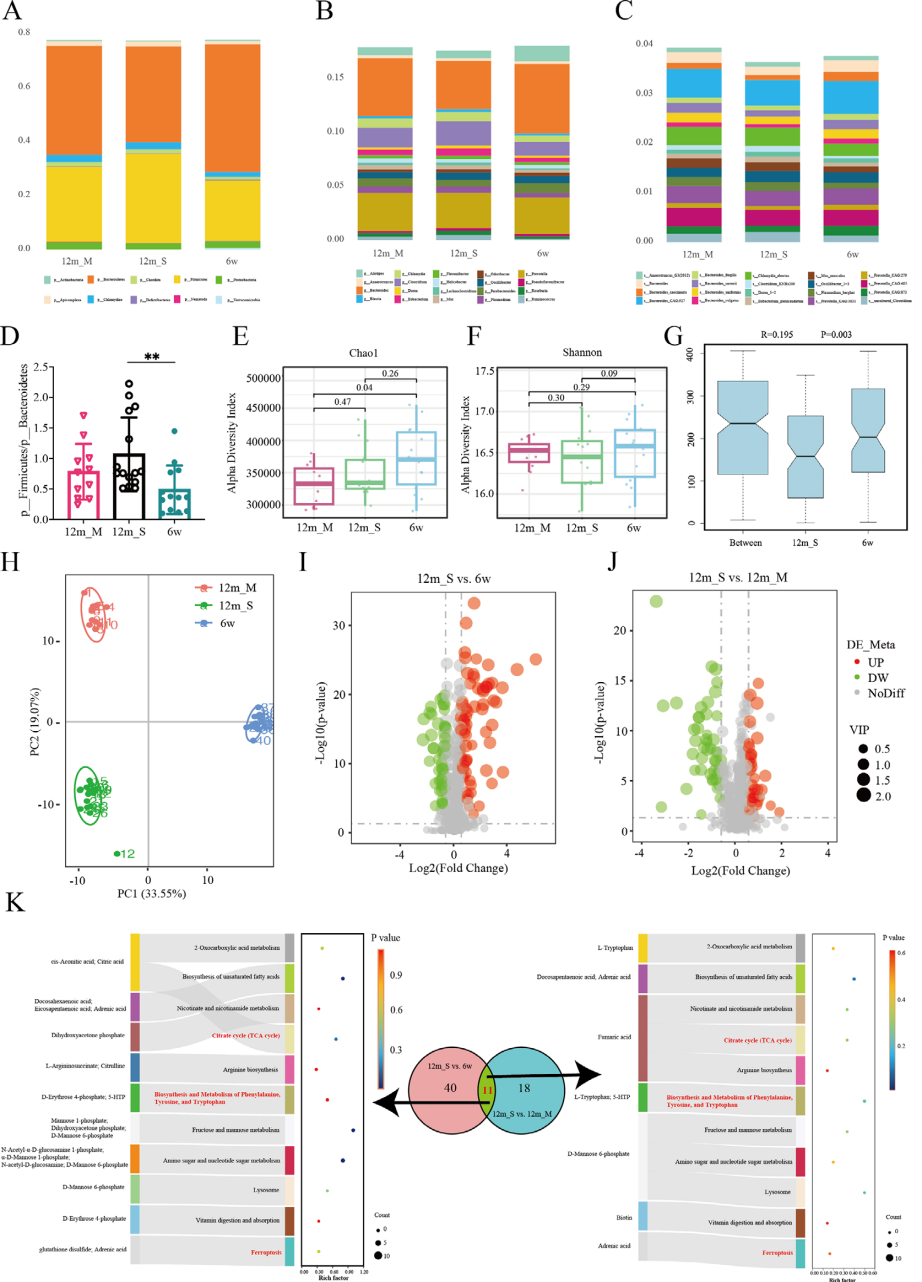
Identification of alterations in the gut microbial composition and cochlear metabolites across groups. (A) The relative abundances of microbiota at the phylum level in fecal samples from different groups. (B) The relative abundances of microbiota at the genus level in fecal samples from different groups. (C) The relative abundances of microbiota at the species level in fecal samples from different groups. (D) F/B ratios in the different groups (*n* = 15 for the 12m_S group, *n* = 14 for the 12m_M group, and *n* = 11 for the 6w group). **p < 0.01. One‐way ANOVA. (E) Chao1 indices of the different groups (*n* = 15 for the 12m_S group, *n* = 14 for the 12m_M group, and *n* = 11 for the 6w group). One‐way ANOVA. (F) Shannon indices of the different groups (*n* = 15 for the 12m_S group, *n* = 14 for the 12m_M group, and *n* = 11 for the 6w group). One‐way ANOVA. (G) The bacterial communities were assessed on the baisis of Bray–Curtis distances (12m_S vs. 6w). (H) PCA model showing significant differences in the cochlear metabolomic profiles among the different groups. (I) Volcano plot‐of the DAMs identified between the 12m_S and 6 w groups. (J) Volcano plot of DAMs identified between the 12m_S and 12m_M groups. (K) KEGG‐based functional characterization of the DAMs, highlighting pathways commonly enriched across the two comparison groups (ARHL‐associated pathways are marked in red).

Building on the findings in Section 2.2 implicating changes in the gut microbiota in the progression of ARHL, we next explored the relationships between the gut microbiota in different groups of mice at different ages and with hearing thresholds via metagenomic approaches. At the phylum level, the composition of the gut microbiota in the three groups was dominated by Bacteroidetes and Firmicutes, whose average relative abundances were 47% and 28%, respectively (Figure 4A). In adults, Firmicutes typically dominate the gut, followed by Bacteroidetes, and the ratio of Firmicutes to Bacteroidetes (F/B) is a crucial biomarker of intestinal homeostasis [6, 7]. Kondo et al. reported that fructooligosaccharides (FOSs), which are nondigestible oligosaccharides and prebiotics, could alter the F/B ratio (via an increase in the abundance of Bacteroidetes and a decrease in the abundance of Firmicutes), promoting SGN survival and decreasing oxidative stress [22]. Monica et al. reported that the F/B ratio increased with age and that an aging microbiome may increase the level of systemic proinflammatory cytokines and affect the outcome of age‐related diseases [23]. Recently, Guo et al. reported that the F/B ratio was significantly higher in a noise‐exposed group than in a control group in vivo [14]. We found that the F/B ratio increased with age and hearing threshold (6w, 0.49; 12m_M, 0.78; 12m_S, 1.07) (Figure 4D). However, the difference between the 12m_M and 12m_S groups was not statistically significant. Furthermore, the microbial composition at both the genus and species levels was also analyzed (Figure 4B,C).

The similarity of the bacterial communities was assessed using analysis of similarity (ANOSIM) based on Bray–Curtis distances. ANOSIM revealed significant differences in the gut microbiota between the 12m_S and 6w groups (r = 0.195, *p* = 0.003) (Figure 4G). However, no significant difference was detected between the 12m_S and 12m_M groups (*r* = ‐0.04, *p* = 0.731) (Figure S2A). A comparative analysis of the fecal microbiota using the Wilcoxon rank‐sum test with false discovery rate (FDR) correction and linear discriminant analysis effect size (LEfSe) was conducted to identify specific bacterial taxa linked to ARHL. Notable variations in the gut microbial compositions were observed among the 12m_S, 12m_M, and 6 w groups, ranging from phylum to species classifications (Figure S2C–H).

In microbiology, α diversity refers to the richness and diversity of species within a specific environment, and is typically asssessed using the Chao1 and Shannon indices. However, reports on the relationship between bacterial diversity and aging remain controversial [24, 25]. In this study, both indices were greater in the 6w group than in the 12m groups (Figure 4E,F). Furthermore, compared with the 12m_M group, the 12m_S group presented lower richness and diversity (Figure 4F). These findings may indicate that as age and hearing thresholds increase, the richness and diversity of the gut microbiota decrease. Although several comparisons did not reach statistical significance, the observed trends may suggest correlations between gut microbiota richness and diversity and ARHL.

Given the significant between‐group differences between 12m_S and 6w identified by ANOSIM, we performed a Kyoto Encyclopedia of Genes and Genomes (KEGG) pathway enrichment analysis to explore the potential biological functions of the differentially abundant microbiome features. Representative reaction pathways were manually constructed on the basis of KEGG pathway maps. Accordingly, 157 KEGG pathways (level 3) were identified (Table S1), including 76 pathways classified under metabolism, 25 classified under organismal systems and 23 classified under human diseases. Metabolism‐related mechanisms such as lipid metabolism, metabolism of cofactors and vitamins, carbohydrate metabolism, the endocrine system and signal transduction were the most abundant pathways at level 2. These findings suggest that the gut microbiota may mediate downstream phenotypic changes via metabolic pathways. Therefore, we next performed a metabolomic analysis of the cochlea.

### 2.4 Alterations in the Composition of Cochlear Metabolites in ARHL

To comprehensively characterize the cochlear metabolic landscape in ARHL, we performed untargeted metabolomic profiling of cochlear tissues from mice across different age and hearing threshold groups. Principal component analysis (PCA) revealed clear separation among the three groups, reflecting distinct metabolite compositions (Figure 4H). We identified 112 differentially abundant metabolites (DAMs) between the 12m_S and 6w groups and 81 DAMs between the 12m_S and 12m_M groups (Figure 4I,J). The majority of these DAMs were organic acids and derivatives, as well as lipids and lipid‐like molecules (Figure S3A). Volcano plot analysis further highlighted DAMs, with clear clusters of upregulated and downregulated DAMs (Figure 4I,J).

Given the many DAMs identified in both comparison groups (12m_S vs. 6w and 12m_S vs. 12m_M), we performed KEGG pathway enrichment analysis and focused on pathways common to both (Figure 4K). Eleven pathways were co‐enriched including phenylalanine, tyrosine, and tryptophan metabolism, the TCA cycle, and ferroptosis, among others. Notably, pathways (shown in red), namely, Biosynthesis and metabolism of phenylalanine, tyrosine, and tryptophan, the TCA cycle, and ferroptosis, have been previously linked to ARHL pathogenesis, indicating that metabolic reprogramming within these pathways may be a core mechanism underlying ARHL progression from early predisposition to advanced functional decline.

The TCA cycle, a central pathway in energy metabolism, was enriched in key intermediates, including cis‐aconitate and citrate. Dysregulation of this pathway may play a critical role in ARHL due to the high energy demand of cochlear cells and their dependence on mitochondrial oxidative phosphorylation [26, 27, 28, 29]. Ferroptosis, an iron‐dependent regulated cell death process, was also enriched, with increasing evidence underscoring its involvement in sensorineural hearing loss (SHL) and ARHL and its contribution to cochlear cell damage [30, 31]. Notably, perturbations in the biosynthesis and metabolism of phenylalanine, tyrosine, and tryptophan, pathways that interconnect neuromodulation, oxidative stress, and amino acid metabolism, were observed. Our DAM analysis revealed the enrichment of key intermediates such as 5‐HTP, D‐erythrose 4‐phosphate, and L‐tryptophan. These alterations suggest the disruption of neurotransmitter equilibrium, aggravated oxidative injury, and compromised protein homeostasis [32, 33, 34].

Collectively, our metabolomic profiling results reveal substantial alterations in the cochlear metabolic landscape across different ages and hearing thresholds. These disturbances likely underlie critical pathological processes in ARHL.

### 2.5 Proteomics‐Based Identification of the PI3K/Akt Pathway as a Key Mediator Involved in ARHL

**FIGURE 5:**
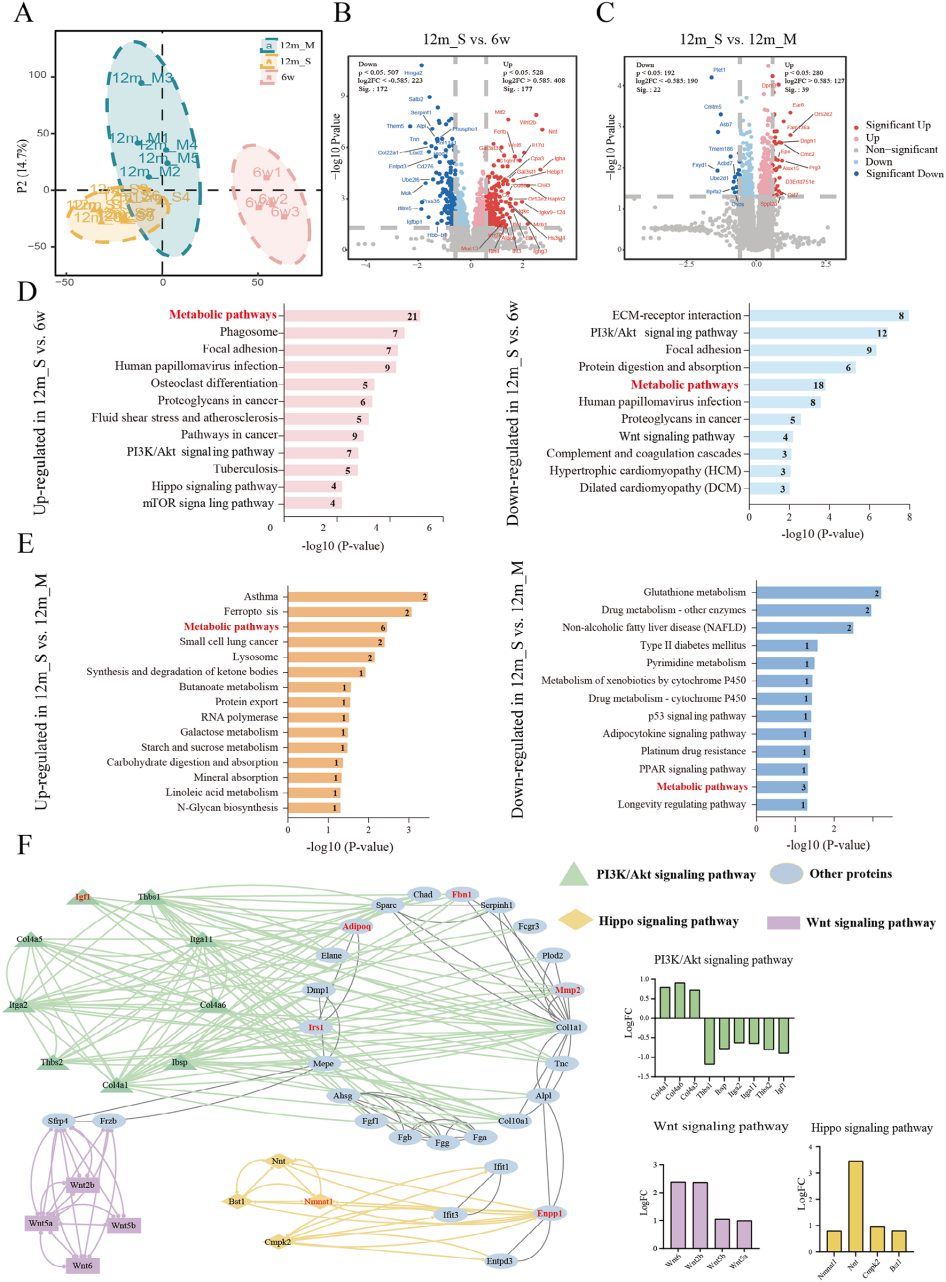
Cochlear proteomic profiles across groups. (A) PLS‐DA model showing the significant discrimination of different groups based on the cochlear proteomic profile. (B) Volcano plot of DEPs identified between the 12m_S and 6w groups. (C) Volcano plot of DEPs identified between the 12m_S and 12m_M groups. (D) Overview of KEGG terms related to molecular functions in the 12m_S and 6w comparison groups. (E) Overview of KEGG terms related to molecular functions in 12m_S and 12m_M comparison groups. (F) PPI network of cochlear DEPs in the comparison of the 12m_S and 6w groups (targets associated with metabolic pathways are marked in red).

Partial least squares discriminant analysis (PLS‐DA) revealed clear separation among the three groups (Figure 5A). Proteomic differences across age groups and hearing‐threshold categories were analyzed and are presented as volcano plots (Figure 5B,C), highlighting significantly differentially expressed proteins (DEPs). In the 12m_S and 6w groups, 172 proteins were downregulated, and 177 were upregulated. In contrast, the comparison of the 12m_S and 12m_M groups revealed only 22 downregulated and 39 upregulated proteins. KEGG enrichment analysis was conducted to identify potential pathways linked to these DEPs (Figure 5D,E). Notably, metabolic pathways were significantly enriched in both comparisons. Metabolic pathways typically comprise an interconnected network of metabolites and enzymes. Changes in the proteome may reflect adaptive responses within the entire metabolic network. Alterations in the gut microbiota could lead to the metabolic reprogramming of cochlear cells, promoting the activation of specific metabolic pathways. This reprogramming may result in altered protein expression patterns, leading to the observed enrichment of metabolic pathways.

Specifically, comparison of the 12m_S and 6w groups led to the identification of a substantial number of DEPs. Thus, a protein–protein interaction (PPI) network was constructed to further investigate important target interactions (Figure 5F). Hubs were identified on the basis of betweenness centrality (BC) scores using Cytoscape. In network analysis, the BC score serves as a critical metric for assessing the importance of nodes within a network. Specifically, the BC value indicates the extent to which a node acts as a bridge among other nodes. A higher BC value indicates that a node plays a stronger intermediary role in connecting other nodes [35]. Additionally, the nodes associated with metabolic pathways are highlighted in red within the network diagram. We identified relevant proteins associated with the PI3K/Akt pathway, such as Thbs1, Itga2, Thbs2, Ibsp, Col4a5, Col4a1, Itga11, and Col4a6. Additionally, proteins related to the Hippo pathway, including Nnt, Bst1, Cmpk2, and Nmnat1, were identified. Components of the Wnt signaling pathway, including Wnt5a, Wnt2b, Wnt6, and Wnt5b, were also located in relatively central positions within the network. Notably, the PI3K/Akt pathway encompasses the greatest number of nodes in the network, and these targets exhibit high BC scores, positioning them at the core of the network. These findings suggest that the PI3K/Akt pathway and its associated targets may represent key factors related to ARHL pathogenesis and should be the focus of future research.

### 2.6 Integrated Multi‐Omics Analysis Identifies Key Targets Associated with ARHL

**FIGURE 6:**
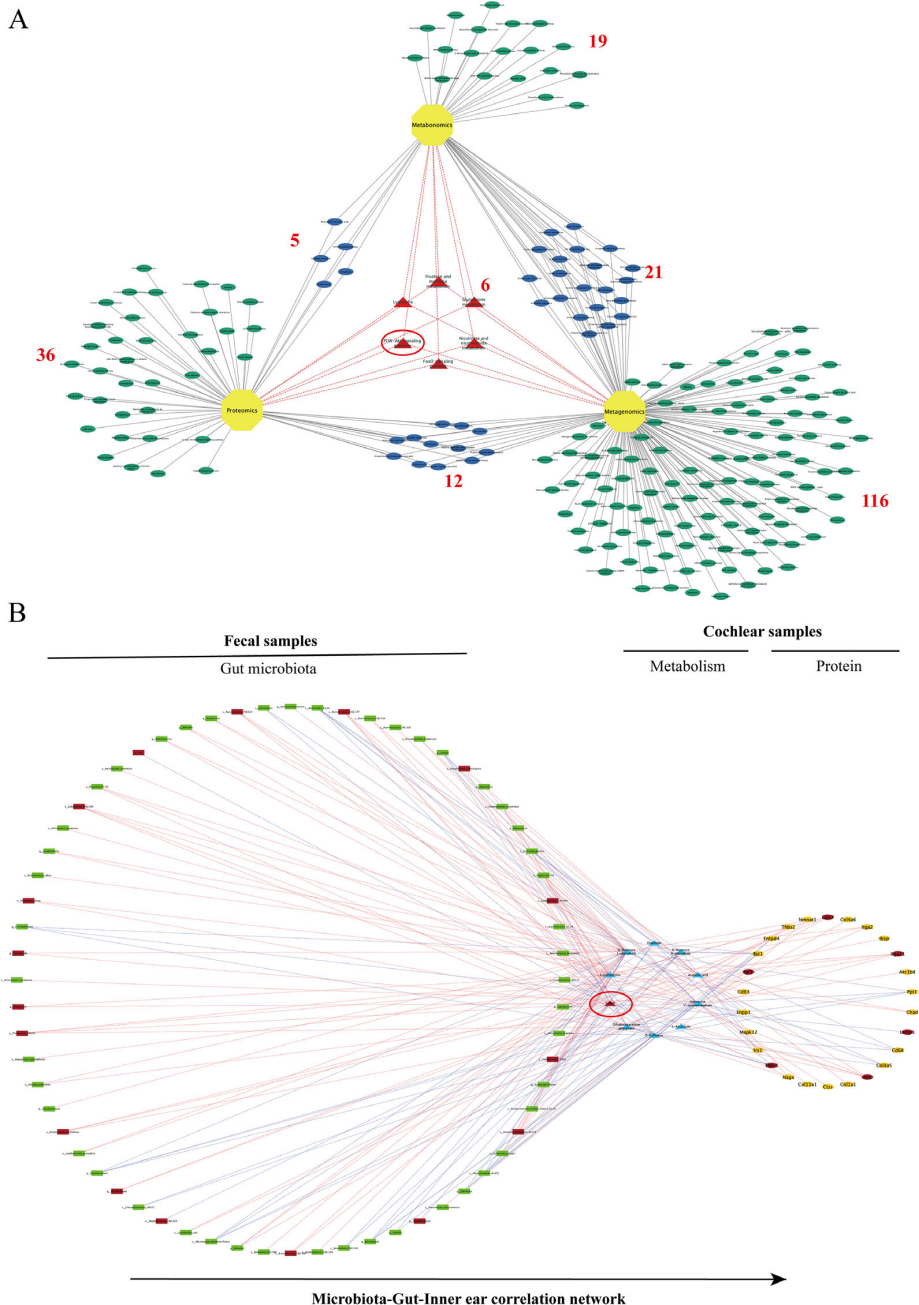
Possible pathways connecting the gut and inner ear in ARHL model mice. (A) Venn diagram showing the commonly enriched KEGG pathways identified from the proteomic, metabolomic, and metagenomic analysis upon comparison of the 12m_S and 6w groups. (B) Correlation network of differentially abundant gut microbiome taxa and DAMs and DEPs in the cochlea (targets associated with 5‐HTP are marked in red).

Given the significant differences observed between the 12m_S and 6w groups across multiple omics layers, particularly at the metagenomic level, an integrated network analysis was conducted. This analysis focused on the differentially abundant molecules identified in the proteomic, metabolomic, and metagenomic data to explore common mechanisms of ARHL that involve gut microbes, functional metabolites, and protein molecules (Figure 6). The associations between the gut microbiome taxa and metabolites and between metabolites and proteins were evaluated using Spearman's rank correlation analysis. In the correlation analysis, 349 gut microbes, 61 metabolites and 289 protein molecules were considered. A total of 6071 statistically significant pairs were identified between the gut microbiome taxa and the cochlear metabolites. A total of 8270 significant correlations  were identified between the cochlear metabolites and protein molecules. The correlation data are clustered and visualized in the heatmap shown in Figure S4.

Furthermore, we identified the enriched KEGG pathways across the three omics datasets by constructing a Venn diagram (Figure 6A). Notably, six pathways were identified as common across these datasets: nicotinate and nicotinamide metabolism, the FoxO signaling pathway, the PI3K/Akt signaling pathway, fructose and mannose metabolism, the lysosome pathway, and glutathione metabolism. These findings suggest that the intersecting pathways may play pivotal roles in the shared mechanisms across the three omics layers, potentially serving as core mechanisms mediating ARHL through the gut microbiota. Consequently, molecular targets associated with these pathways at both the proteomic and metabolomic levels were systematically screened using manual and automated methods. This analysis was followed by a correlation analysis with the most significantly differentially abundant microbial taxa at the genus and species levels to construct a correlation network. A network including 64 microbial taxa, 10 DAMs and 26 DEPs was constructed, suggesting that these differentially abundant molecules might act as vital bridges between the gut and inner ear (Figure 6B).

Specifically, the metabolites and their associated mechanisms serve as crucial hubs and bridges that significantly enrich and correlate the differentially abundant gut microbes and DEPs in the cochlea, potentially playing vital roles in ARHL mediated by the gut microbiota. This metabolomic approach integrates insights from both metabolic and functional perspectives, elucidating the complex interactions and regulatory relationships between the gut microbiota and host protein dynamics. Among the metabolites analyzed, the levels of adenine 5'‐monophosphate, 5‐HTP, D‐mannose 1‐phosphate, ascorbic acid, and D‐raffinose were strongly correlated with the abundance of differentially abundant microbial taxa and the levels of DEPs. Notably, 5‐HTP emerged as the most compelling candidate. First, the level of 5‐HTP was significantly lower in the 12m_S group than in the 6w group (Figure S3B). Furthermore, 5‐HTP exhibited extensive connectivity within the interaction network, indicating strong interactions with numerous other targets (Figure 6B).

The abundance of numerous species of Firmicutes was found to be associated with 5‐HTP levels (Figure 6B). Notably, the abundance of Brachyspira exhibited a strong negative correlation with 5‐HTP levels (Spearman's ρ = ‐0.67). Additionally, the abundances of various Ruminococcus species, including Ruminococcus_CAG:403, Ruminococcus_CAG:177, and Ruminococcus_FC2018, were associated with 5‐HTP levels. Among these, the abundance of Ruminococcus_CAG:177 showed the strongest negative correlation with the 5‐HTP levels (Spearman's ρ = −0.80). Moreover, the abundance of Ruminococcus was strongly negatively correlated with the levels of bone formation factors, gut barrier indicators, and the bone volume fraction but strongly positively correlated with the levels of bone resorption factors and intestinal inflammatory factors in an in vivo study [36]. Additionally, Coprobacillus abundance was negatively associated with 5‐HTP levels (Spearman's *ρ* = ‐0.65) and has been reported to trigger gut inflammation and the loss of intestinal barrier integrity in patients consuming a high‐fat and low‐cellulose diet [37, 38]. Additionally, through the integration of findings from proteomic analyses in section 2.5, the PI3K/Akt pathway was identified as potentially the most critical pathway in ARHL. Notably, several proteins significantly associated with 5‐HTP (Figure 6B), including Igf1, Lamp1, Itga11, and Thbs1, play important roles in the PI3K/Akt signaling cascade.

As detailed in Section 2.4, the biosynthesis and metabolism of phenylalanine, tyrosine, and tryptophan represent one of the key pathways im in ARHL. Tryptophan, an essential amino acid, is metabolized in a gut microbiota‐dependent manner and serves as the direct precursor for 5‐HTP. Under physiological conditions, 5‐HTP, but not serotonin (5‐HT), can cross the BBB, suggesting its potential role in central nervous system regulation [39]. These characteristics have attacted significant research attention, particularly in the context of gut–brain axis signaling. Certain probiotic strains, including Lactobacillus and Bifidobacterium, have been shown to modulate central nervous system‐related disorders and behaviors through the regulation of 5‐HTP synthesis [40, 41]. Notably, Bifidobacterium was identified in our metagenomic analysis as exhibiting significant intergroup differences in abundance, with its levels markedly decreasing as mice aged and hearing thresholds increased (Figure S2B, S2F). A healthy gut microbiome may thus optimize tryptophan utilization, thereby supporting 5‐HTP production. Accumulating evidence continues to underscore the systemic protective roles of 5‐HTP, which are mediated primarily through anti‐inflammatory and antioxidant mechanisms, and several in vitro studies have reported that the gut microbiota of both rats and humans synthesize 5‐HTP, highlighting the multifaceted role of 5‐HTP in health and disease [39, 42, 43]. In contrast, metabolites such as oleamide, D‐mannose 1‐phosphate, D‐mannose 6‐phosphate, and adenosine 5'‐monophosphate can be influenced by numerous endogenous factors, including hormones, organ function, and energy metabolism [44, 45, 46, 47]. This complexity may hinder our understanding of how the gut microbiota regulates the levels of these metabolites and their effects on ARHL.

### 2.7 5‐HTP Restores the PI3K/Akt Pathway Disrupted by D‐Gal‐Induced Aging

**FIGURE 7:**
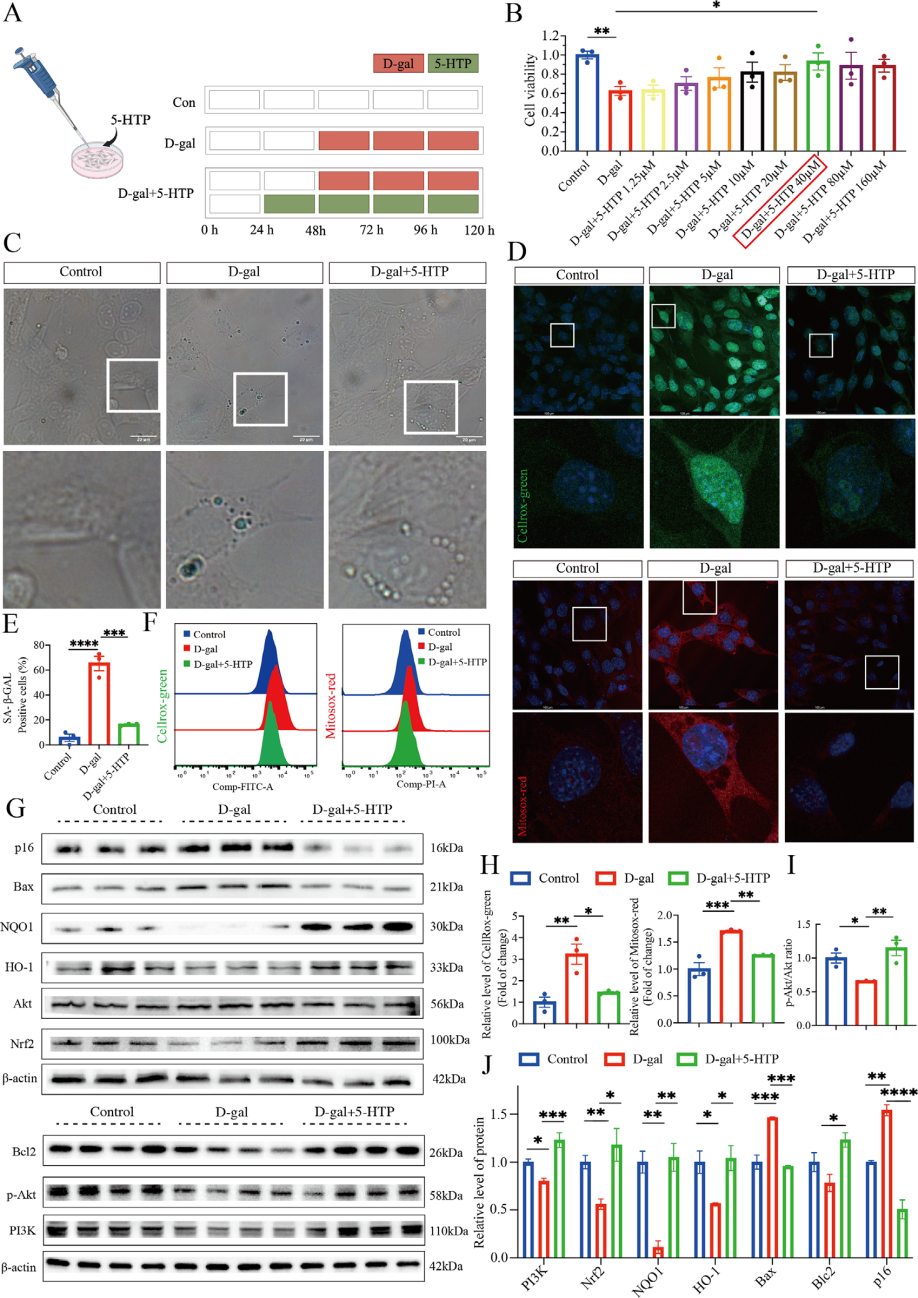
Effects of 5‐HTP on D‐gal‐induced senescence in HEI‐OC1 cells. (A) Treatment of the cellular model with 5‐HTP and D‐gal. (B) Cell viability following treatment with 5‐HTP and D‐gal (*n* = 3). *p < 0.05 and **p < 0.01. Unpaired *t*‐test. (C) SA‐β‐GAL staining in different groups. Scale bar, 20µm. (D) Cellular and mitochondrial ROS levels were assessed by live‐cell staining. Scale bar, 100µm. (E) Quantitative analysis of the data presented in (C) (*n* = 3). ***p < 0.001, ****p < 0.0001. One‐way ANOVA test. (F) Quantitative analysis of the data presented in (D). (G)Expression levels of the target proteins in the different groups. (H) Quantitative analysis of the data presented in (D) (*n* = 3). *p < 0.05, **p < 0.01, and ***p < 0.001. One‐way ANOVA. (I,J) Semiquantitative densitometric analysis of the corresponding blots across the various groups (*n* = 3/*n* = 4). *p < 0.05, **p < 0.01, ***p < 0.001, and ****p < 0.0001. One‐way ANOVA.

We first established a D‐gal‐induced in vitro model of ARHL (Figure S5A) and determined the optimal concentration and treatment duration for 5‐HTP treatment (Figure 7A,B). Given that our integrated multi‐omics analysis identified the PI3K/Akt pathway as a 5‐HTP–associated hub involved in regulating redox homeostasis and ARHL pathogenesis, we next examined whether 5‐HTP modulates this pathway. Western blot analysis revealed that D‐gal treatment significantly suppressed PI3K/Akt pathway activation compared with the control group. Notably, 5‐HTP treatment restored pathway activity, thereby reversing the D‐gal‐mediated suppression (Figure 7G,I,J).

### 2.8 5‐HTP Enhances Nrf2‐Mediated Antioxidant Defenses and Suppresses Oxidative Stress

Proteomic analysis revealed a marked downregulation of the expression of NQO1,a key antioxidant enzyme, in the aged cochlea (Figure S5D). Given that NQO1 and other major antioxidant proteins are transcriptionally regulated by Nrf2, whose activity is critically controlled by the upstream PI3K/Akt pathway [48, 49], we next investigated whether 5‐HTP could modulate the PI3K/Akt‐antioxidant signaling cascade. We found that D‐gal‐induced inhibition of Akt phosphorylation was associated with decreased Nrf2 protein accumulation and reduced expression of its downstream effectors, HO‐1 and NQO1. Importantly, 5‐HTP treatment significantly enhanced the expression of Nrf2, HO‐1, and NQO1 (Figure 7G,J).

To validate the functional consequence of this antioxidant system enhancement, cellular and mitochondrial reactive oxygen species (ROS) levels were assessed using flow cytometry and live‐cell staining with Cellrox‐green and Mitosox‐red. D‐gal treatment triggered a significant increase in both intracellular and mitochondrial ROS accumulation (Figure 7D,F,H). According to the free radical theory of aging, the progressive accumulation of oxidative damage caused by ROS,a natural byproduct of mitochondrial respiration, contributes to age‐related functional decline. While endogenous antioxidant systems are typically sufficient to neutralize ROS under physiological conditions, aging leads to an imbalance in which ROS production ultimately exceeds the cell's detoxifying capacity, causing mitochondrial dysfunction and DNA damage. This persistent oxidative stress is a major contributor to irreversible hearing loss [50, 51, 52, 53]. Notably, 5‐HTP administration effectively attenuated ROS accumulation and re‐established cellular redox homeostasis (Figure 7D,F,H).

### 2.9 5‐HTP Attenuates Apoptosis and Cellular Senescence

Given that oxidative stress is an important driver of apoptotic cell death in ARHL [54, 55], and our proteomic results also suggested an imbalance in the expression of apoptosis‐related proteins (Figure S5D), we next examined the expression of pro‐ and anti‐apoptotic factors. D‐gal treatment significantly increased the expression of the pro‐apoptotic protein Bax at both mRNA (Figure S5B) and protein levels (Figure 7G,J), while the level of the anti‐apoptotic protein Bcl2 showed a decreasing trend. Notably, 5‐HTP intervention successfully reversed this trend (Figure 7G,J; Figure S5B,C). We then assessed the expression of markers of cellular senescence. Compared with D‐gal treatment alone, 5‐HTP treatment significantly reduced the expression of p16 (Figure 7G,J), a cyclin‐dependent kinase inhibitor that enforces permanent cell cycle arrest, and decreased the proportion of SA‐β‐gal‐positive cells (Figure 7C,E).

## 3 Discussion

With the rapidly aging global population, ARHL has emerged as a critical health challenge that severely affects quality of life of older adults [1, 2, 3]. The pathogenesis of ARHL remains incompletely elucidated, and safe, effective pharmacological interventions are still lacking in clinical practice [4]. In recent years, the integration of animal models and FMT has opened new avenues for investigating the role of the gut microbiota in age‐related disorders, particularly neurodegenerative conditions and sensory system dysfunctions [56, 57]. In this study, using a GF mouse model, we systematically evaluated the effect of the gut microbiota on the development and progression of ARHL by transplanting the gut microbiota from donors of varying ages with varying hearing levels and performed comprehensive analyses.

Although no effective pharmacological therapies are currently available for the treatment of ARHL, several preclinical studies have explored the potential of compounds such as selegiline, rapamycin, melatonin, and aspirin to delay its progression [17, 58, 59, 60, 61]. However, some of these studies have failed to present robust auditory outcome data [58, 61], with many reporting only marginal differences in hearing thresholds following pharmacological intervention [17, 59, 60]. Furthermore, the clinical translation and long‐term application of these candidate therapies are limited by their narrow therapeutic window, dose‐dependent toxicity, and a range of adverse effects, including metabolic disturbances, depression, insomnia, and gastrointestinal mucosal damage [62, 63, 64, 65]. In our study, the differences in hearing thresholds between the FMT‐6w and FMT‐12m_S groups reached 17.33dB at click, 14.03dB at 4kHz, 22.08dB at 8kHz, and 4.92dB at 16kHz. In addition, microbiome‐based interventions, such as the administration of probioticsor prebiotics and FMT have attacted attention due of their favorable safety profile and food‐grade status, making them particularly suitable for long‐term health management in elderly individuals [66, 67, 68]. Our results demonstrate that FMT not only significantly reduced cellular senescence and preserved structural integrity in cochlear tissues but also provided broader benefits for overall systemic health. These observations further underscore the systemic safety of FMT and support the clinical translatability of microbiota‐targeted strategies.

On the basis of these findings and the supporting literature, we propose the following hypothesis: FMT from young donors reshapes the host systemic metabolome, thereby enhancing the production and release of key metabolites such as 5‐HTP. These metabolites may enter the systemic circulation and cross the BLB, subsequently modulating ARHL progression through multiple mechanistic pathways. Our integrative multi‐omics analysis identified 5‐HTP as a pivotal metabolite linking gut microbiota composition to cochlear health. Drawing from our experimental results and the literature, we propose a mechanistic pathway in which the alterations in the abundance of specific gut microbial taxa, including Ruminococcus and Coprobacillusmay alleviate intestinal inflammation and improve intestinal barrier integrity [35, 36, 37], thereby optimizing the gut microenvironment and promoting Bifidobacterium proliferation. Bifidobacterium may subsequently modulate tryptophan metabolism, leading to increased 5‐HTP biosynthesis [39, 40]. Upon reaching the cochlea via the systemic circulation, 5‐HTP activates the PI3K/Akt‐antioxidant signaling axis, enhancing antioxidant defenses and effectively suppressing ROS production and cellular apoptosis‐related dysregulation，thereby reducing cellular senescence. Moreover, our proteomic analysis revealed the upregulation of the expression of multiple pro‐inflammatory factors in the aged cochlea (Figure S5D), while 5‐HTP treatment significantly reduced Nfkb1 mRNA expression (Figure S5E). Nfkb1 encodes a subunit of the NF‐κB complex, a master regulator of inflammation. Together, these data may support antioxidant and anti‐inflammatory effects of 5‐HTP in the cochlea. This mechanistic perspective aligns with recent findings based on a primate single‐nucelus transcriptomic altas from Liu's team, which identified neuroinflammation as one of the hallmark features of cochlear aging [9]. Collectively, these results underscore 5‐HTP as a key microbiota‐regulated metabolic hub in mitigating ARHL. However, the upstream molecular mechanisms governing 5‐HTP‐mediated regulation of the PI3K/Akt pathway warrant further investigation. We hypothesize that multiple mechanisms may converge in this process, including direct modulation of PI3K/Akt activation through antioxidant activity that reduces ROS levels; effects on cellular metabolism and energy sensing pathways; and given the presence of 5‐HT receptors in the auditory system [69, 70], local conversion of 5‐HTP to 5‐HT, which could trigger subsequent receptor‐dependent PI3K recruitment and Akt phosphorylation [71, 72]. These mechanisms likely act synergistically to produce the protective effects observed in our study.

Our work advances the current understanding in several respects. First, to our knowledge, this is the first study to directly demonstrate the causal relationship between the gut microbiota composition and ARHL progression using GF mice and FMT. Second, our integrated multi‐omics approach provides unprecedented molecular insights into the gut‐inner ear axis, identifying specific gut microbial taxa, metabolites, and signaling pathways involved in cochlear aging. Third, the magnitude of hearing improvement observed following FMT, together with a favorable safety profile, underscores the translational potential of microbiome‐based strategies. From a clinical perspective, our results suggest that multi‐omics profiling could facilitate the development of noninvasive diagnostic tools and personalized intervention strategies based on gut microbiome signatures, providing precise risk stratification and targeted preventive measures for high‐risk populations. Moreover, microbiome‐targeted interventions (such as probiotics, prebiotics, or FMT) hold promise as safe and effective avenues for preventing or treating ARHL and represent a key direction for future research.

This study has several limitations that should be addressed in future work. (1) Although previous studies have shown that Bifidobacterium and Lactobacillus can modulate tryptophan–5‐HTP metabolism to influence central nervous system disorders via the gut–brain axis [40, 41] and in vitro evidence has indicated that the gut microbiota from both rats and humans can directly produce 5‐HTP [39, 42, 43], the causal relationships and detailed mechanisms by which specific species identified in this study cooperatively or competitively regulate tryptophan–5‐HTP metabolism through the gut–inner ear axis remain unclear. Future studies should employ mono‐colonization experiments to establish direct causality and elucidate the underlying regulatory mechanisms. (2) Although prebiotic and probiotic interventions have shown favorable safety profiles in broad clinical contexts [66, 68], their application in otology remains nascent. Our study employed C57BL/6J mice and HEI‐OC1 cells—the most widely used and well‐characterized models in ARHL research [58, 73, 74, 75].Nevertheless, clinical translation will require validation across additional disease‐relevant inner ear cell types and multicellular systems, as well as long‐term functional efficacy and safety assessments in primate models. Future work will leverage cochlear organoids and organotypic explants to capture multicellular interactions and lateral‐wall physiology, and will integrate spatial multi‐omics analyses with single‐cell sequencing data to generate cell type–resolved, spatially contextualized maps of microbiota‐mediated aging pathways within the inner ear. (3) While grip strength assessment provides a valuable preliminary functional readout, this single measure is insufficient to comprehensively capture the complexity of muscle metabolism, repair, and overall functional status. Future studies should adopt a more integrated assessment framework, incorporating measures such as leg press strength, gait analysis, muscle mass, and omics profiling, to more fully elucidate the broad impact of the gut microbiota on systemic health and age‐related decline. (4) When the 12m_M and 12m_S groups were compared, the differences in the F/B ratio and bacterial community similarity showed a trend but did not reach statistical significance. This may be because, unlike in human studies, even the best‐hearing 12m C57BL/6J mice exhibit mild‐to‐moderate hearing loss, particularly at high frequencies. This baseline pathology may limit the discernible divergence in these broad, aggregate microbiota metrics. Interestingly, analyses of the abundances of specific taxa revealed numerous differentially abundant bacteria between the two groups (Figure 4A–C; Figure S2C,F,H). This suggests that specific bacterial taxa or their functions—rather than overall community similarity—may be more sensitive indicators of auditory performance within the same age cohort. (5) Our multi‐omics dataset revealed additional metabolites, such as adenine 5'‐monophosphate, D‐mannose 1‐phosphate, ascorbic acid, and D‐raffinose, whose levels strongly correlate with multiple components of the integrative network (Figure 6B). These metabolites may serve as key gut‐derived or microbiota‐regulated hub molecules, and further investigation of their roles in ARHL pathogenesis and therapeutic potential are warranted.

In summary, this study demonstrates the combined use of FMT in GF mouse models to directly assess the effects of the gut microbiota from mouse donors of varying ages and hearing levels on ARHL progression. Our findings provide a comprehensive characterization of the molecular landscape underlying ARHL, establishing a high‐value multi‐omics dataset for future research. Notably, integrative analyses suggest that 5‐HTP is a microbiota‐linked metabolic hub that may mediate gut microbiota–driven changes during ARHL progression and represents a promising therapeutic target. Together, these findings support the development of strategies that modulate the gut microbiota and its metabolic network to alleviate ARHL in clinical settings.

## 4 Experimental Section

### 4.1 Experimental Animals

All animal experiments and procedures conducted in this study were performed in strict adherence to the ethical guidelines outlined in the eighth edition of the Guide for the Care and Use of Laboratory Animals published by the National Research Council (NRC). Furthermore, these practices were approved by the Institutional Animal Care and Use Committee (IACUC), ensuring compliance with the standards of animal welfare and ethical research (Nos. 202410032Z and GPTAP20240920‐1). The mice were housed under optimal conditions, maintained at a temperature of 22 ± 1°C, and exposed to a regulated 12:12h light–dark cycle, with unrestricted access to food and water.

### 4.2 Study Design

Wild‐type male C57BL/6J and GF mice were purchased from GemPharmatech (Nanjing, China). All the mice were randomly allocated into three groups according to their hearing threshold [21]: the 6w group, the 12m_M group, and the 12m_S group. GF mice were selected for FMT. 12m male GF mice were randomly divided into three groups: the control group, FMT‐12m_S group, and FMT‐6w group. After three months of FMT intervention, endpoint data were collected.

### 4.3 ABR Measurement

The ABR test was performed to measure the hearing thresholds of the mice. Details of the ABR test procedure have been provided previously [76]. Suprathreshold amplitudes and latencies of wave I were assessed at 8kHz to evaluate age‐related auditory nerve degeneration. The wave I amplitude (peak‐to‐trough) was measured at 90dB SPL, and latency was defined as the time from stimulus onset to the peak.

### 4.4 Histological Immunofluorescence Staining

All the temporal bones of the experimental mice were harvested, rinsed, and incubated with 4% formaldehyde for 2h at room temperature (RT), followed by decalcification with 10% EDTA for 24h. The cochleae were then dissected into three pieces (apex, middle, and base turns). The tissues were permeabilized with 1% Triton X‐100 for 2h and blocked with 10% donkey serum (D9663; Sigma) for 2h at room temperature, followed by incubation with primary antibodies against MYO7A (1:500 dilution, Proteus BioSciences, 25–6790), CtBP2 (1:500 dilution, BD Biosciences, 612044) and NF (1:500 dilution, Sigma, AB5539) for 24h, after which the corresponding Alexa Fluor‐conjugated secondary antibodies were applied for observation. Nuclei were labeled with DAPI (1:1,000 dilution; Yeasen, 36308ES11). Fluorescence images were obtained with a laser scanning confocal microscope (Leica TCS SP8) using a 40× objective and analyzed with Adobe Photoshop and ImageJ. For the quantification of HCs, MYO7A‐positive HCs (and DAPI‐labeled nuclei) were randomly counted in each turn from three independent cochleae. Fixed regions of five IHCs were randomly selected from three independent cochleae to quantify the number of presynaptic ribbons.

### 4.5 H&E Staining

Samples of cochlear tissue were initially fixed with 4% paraformaldehyde overnight at 4°C, followed by decalcification with 10% EDTA for48 h and dehydration with 15% sucrose and 30% sucrose separately for 24h. The samples were embedded in optimal cutting temperature (OCT) compound and stored at −80°C. The cochlear samples were cut into approximately 10‐µm‐thick sections with a cryostat and then stained with H&E according to the provided instructions. In H&E ‐stained sections, the SV appears as an epithelial strip on the lateral wall of the cochlear duct, nestling between the spiral prominence and Reissner's membrane [77]. Three images showing different regions of the SV (apex, middle, and base turns) were captured randomly under a microscope (Nikon, Tokyo, Japan). The number of SGNs within each group was counted by randomly selecting a fixed size range in three independent H&E‐stained sections under the same magnification.

### 4.6 SA‐β‐GAL Staining

SA‐β‐GAL accumulation was detected using a commercial kit (C0602; Beyotime, Shanghai, China) according to the manufacturer's protocol.

### 4.7 FITC‐Dextran Assay for Intestinal Integrity

Intestinal integrity was determined using a FITC‐dextran assay. Briefly, 300mg/kg FITC‐dextran (75mg/ml in ddH_2_O, DX‐4000‐FITC; Sigma) was administered by oral gavage to each mouse. After 60min, blood was collected from the retro‐orbital plexus under pentobarbital anesthesia and centrifuged (12000 × g at 4°C) for 10min. Diluted serum was added to a 96‐well microplate in duplicate. The concentration of FITC in the serum was determined using a fluorescence spectrophotometer (Synergy H1, BioTek, Winooski, VT) with an excitation wavelength of 485nm and an emission wavelength of 535nm.

### 4.8 Grip Strength Assessment

The forelimb strength of the mice was measured with a grip strength assay (BioSEB G3) according to the manufacturer's instructions.

### 4.9 Sample Collection and Preparation

The C57BL/6J mice were sacrificed by cervical dislocation, and samples of excrement, blood and cochleae were collected from the C57BL/6J mice in each group. Fresh excrement from the digestive tract was immediately collected in EP tubes. Individual mouse serum samples were collected from the animals in serum separation tubes. The removed cochleae were rinsed with PBS, the surrounding excess tissue was peeled off under a microscope, and the sample was quickly placed in liquid nitrogen for storage.

### 4.10 Sample Extraction for Proteomic Analyses

LC–MS/MS analysis was subsequently performed following the homogenization and lysis of fresh cochlear tissues. Mass spectrometry data were analyzed with DIA‐NN software (version 1.8). Volcano plots was constructed to highlight significantly DEPs. PLS‐DA was performed to visualize group separation and feature contributions. KEGG pathway enrichment analysis was conducted using the DAVID website (https://david.ncifcrf.gov/). A FC >1.5 or < 0.67 and *p* < 0.05 were established as the statistical thresholds for identifying proteins with significant differential expression. The relationships among the DEPs were retrieved from STRING and clustered according to the BC score from Cytoscape. The nodes are color‐coded for categorization into three typical signaling pathways associated with ARHL.

### 4.11 Sample Extraction for the Metabolomic Analysis

The cochlear samples were finely ground in liquid nitrogen, resuspended in 80% methanol, incubated, centrifuged, diluted, and subjected to LC–MS/MS for metabolite identification. PCA and univariate analysis were employed to identify DAMs. Specifically, metabolites with VIP > 1.0, FC >1.5 or < 0.67 and *p* < 0.05 were deemed to be differentially abundant. Volcano plots were constructed to visualize and identify the metabolites of interest. Metabolites and metabolic pathways were analyzed with the KEGG database. The enrichment of metabolic pathways was determined based on a ratio criterion and a significance threshold of a *p* value < 0.05.

### 4.12 DNA Extraction and Metagenomic Data Analysis

A metagenomic sequencing assay was performed on fecal samples at SeqHealth Technology Co., Wuhan, China. PCR products spanning 200–500 base pairs in length were selectively enriched, quantified, and sequenced using a NovaSeq 6000 sequencer (Illumina) and the PE150 platform. The abundances of species were determined by aggregating the gene abundances linked to each species from the taxonomic database correlated with the NR library. Furthermore, gene abundance data were used to compute the Shannon and Chao1 diversity indices for each sample. Abundance data were examined across various taxonomic levels. Gene set annotation was performed by alignment with the KEGG database using DIAMOND (version 0.9.10). Alignments of homologous protein sequences for functional annotation were performed against the NR database using DIAMOND. Species classification information was derived from the Greengenes database.

### 4.13 Integrated Network Analysis of Multi‐omics Data

The integration of the metagenomic, metabolomic and proteomic data was performed through correlation analysis. The correlations were computed and processed in R, leveraging the corr function to calculate the Spearman correlation coefficients for all potential pairs of matrices. The statistical significance of pairwise correlations was subsequently determined after adjusting for the FDR using the Benjamini–Hochberg procedure implemented in the Hmisc package in R. An absolute value of the correlation coefficient |ρ| > 0.6 (*p*< 0.05) indicates a statistically significant relationship. Significant correlations were imported into Cytoscape 3.8.2 for an intuitive visualization of the correlation network.

### 4.14 HEI‐OC1 Cell Culture

The HEI‐OC1 cell line was cultured in high‐glucose DMEM (Gibco) supplemented with 5% fetal bovine serum (FBS) (Gibco) in a controlled environment at 33°Cwith 5% CO2.

### 4.15 Drug Administration

HEI‐OC1 cells were pretreated with 40µM 5‐HTP for 24h, after which D‐gal was added together for 72h for subsequent experiments (Figure 7A).

### 4.16 CCK‐8 Assay

Cell viability was evaluated using a CCK‐8 assay (Beyotime). Briefly, HEI‐OC1 cells from each experimental group were washed with prewarmed phosphate‐buffered saline (PBS), followed by the addition of CCK‐8 reagent for a duration of 1.5h of incubation. The absorbance was subsequently measured at 450nm using a microplate reader (Bio‐Rad Laboratories, Hercules, CA, USA). Each experimental condition was assessed in triplicate.

### 4.17 ROS Assay

ROS levels were quantified by Cellrox‐green and Mitosox‐red staining (Thermo Fisher Scientific). Following treatment and washes with phosphate‐buffered saline (PBS), the cells were stained with 5µM Cellrox‐green or 5µM Mitosox‐red in prewarmed serum‐free DMEM in the dark for 40–50min. The fluorescence intensity was subsequently analyzed using a Leica SP8 confocal fluorescence microscope. All the experiments were conducted in triplicate to ensure reproducibility.

### 4.18 Real‐Time PCR

RNA was extracted and subsequently reverse transcribed into complementary DNA (cDNA) with Superscript III reverse transcriptase (Invitrogen). The cDNA was then amplified on an ABI 7500 real‐time PCR system using TB Green (Takara). The GADPH gene served as the endogenous control, and the relative expression levels of the target genes were calculated using the 2^‐ΔΔCT method. The specific primers utilized are detailed in Table S2.

### 4.19 Western Blotting

Total protein was extracted using radioimmunoprecipitation assay (RIPA) lysis buffer according to the manufacturer's guidelines. Equal quantities of protein samples were subjected to sodium dodecyl sulfate–polyacrylamide gel electrophoresis (SDS–PAGE) and subsequently transferred to polyvinylidene fluoride (PVDF) membranes. The membranes were incubated with various primary antibodies. The membranes were then incubated with appropriate secondary antibodies for 2h, after which the protein signals were detected with an enhanced chemiluminescence (ECL) kit (Millipore, Billerica, MA, USA). The following antibodies were used in this study: anti‐rabbit p16 (Abcam, EPR20418), anti‐p‐Akt (Proteintech, 80455‐1‐RR), anti‐Akt (Proteintech, 10176‐2‐AP), anti‐PI3K (Proteintech, 20584‐1‐AP), anti‐Nrf2 (Proteintech, 80593‐1‐RR), anti‐HO‐1 (Proteintech, 81281‐1‐PBS), anti‐NQO1 (Proteintech, 67240‐1‐PBS), ani‐Bax (Proteintech, 60267‐1‐Ig), anti‐Bcl2 (Proteintech, 68103‐1‐PBS), anti‐β‐actin (Proteintech, 81115‐1‐RR), anti‐rabbit IgG (Abcam, 288151), and anti‐mouse IgG (Abcam, 205719). All antibodies were diluted to ratios of 1:500–1:20,000.

### 4.20 Statistical Analysis

GraphPad Prism 8 was used for statistical analyses, and the data are presented as the mean ± SEM. For two independent samples meeting normality and variance assumptions, unpaired *t*‐tests (with Welch's correction as needed) were used; otherwise, the Wilcoxon rank‐sum (Mann–Whitney) test was applied. For multiple groups, one‐way ANOVA was used to assess differences across multiple groups. ANOSIM based on Bray–Curtis dissimilarities was performed in R (vegan package); the ANOSIM R statistic quantifies between‐group separation, and significance was evaluated by permutation testing. P < 0.05 was considered statistically significant. Each experiment was performed at least three times.

## Author Contributions

T.Y., Z.G., H.H., and C.Z. contributed equally to this work. T.Y. conceived the study, carried out the investigation and animal experiments, performed formal analyses and methodology, and, together with W.Y., drafted the original manuscript and revised it. Z.G. performed statistical analyses and animal experiments and contributed to writing and revising the manuscript. H.H. developed the software, performed data visualization, and contributed to writing and revising the manuscript. C.Z. participated in the investigation and contributed to writing and revising the manuscript. W.Y. acquired funding, provided resources and supervision, and wrote the original draft. Y.S. and H.Z. supervised the project, contributed to methodology, and revised the manuscript. Y.T., Q.Q., and H.L. provided additional supervision and methodological guidance and revised the manuscript. J.K. and Z.C. contributed to manuscript revision and validation. M.F. carried out additional investigations and validation. W.Y., Y.S., and H.Z. are the corresponding authors and oversaw the overall direction of the study. All authors discussed the results and approved the final version of the manuscript.

## Conflicts of Interest

The authors declare no conflicts of interest.

## Consent

All the contributing authors have agreed to the publication of this manuscript.

## Supporting information




**Supporting File 1**: advs73974‐sup‐0001‐SuppMat.docx

**Supporting File 2**: advs73974‐sup‐0002‐DataFile.docx

## Data Availability

The data that support the findings of this study are available from the corresponding author upon reasonable request.
