# Supplementary material for: Gut–Metabolome–Proteome Interactions in Age‐Related Hearing Loss: Insights from Fecal Microbiota Transplantation and Multi‐Omics Analyses
Source: Adv Sci (Weinh). 2026 Jan 31;13(18):e14269. doi: 10.1002/advs.202514269 (PMC13042867; doi:10.1002/advs.202514269)

Figure 2C

（1）Apex-12m_S


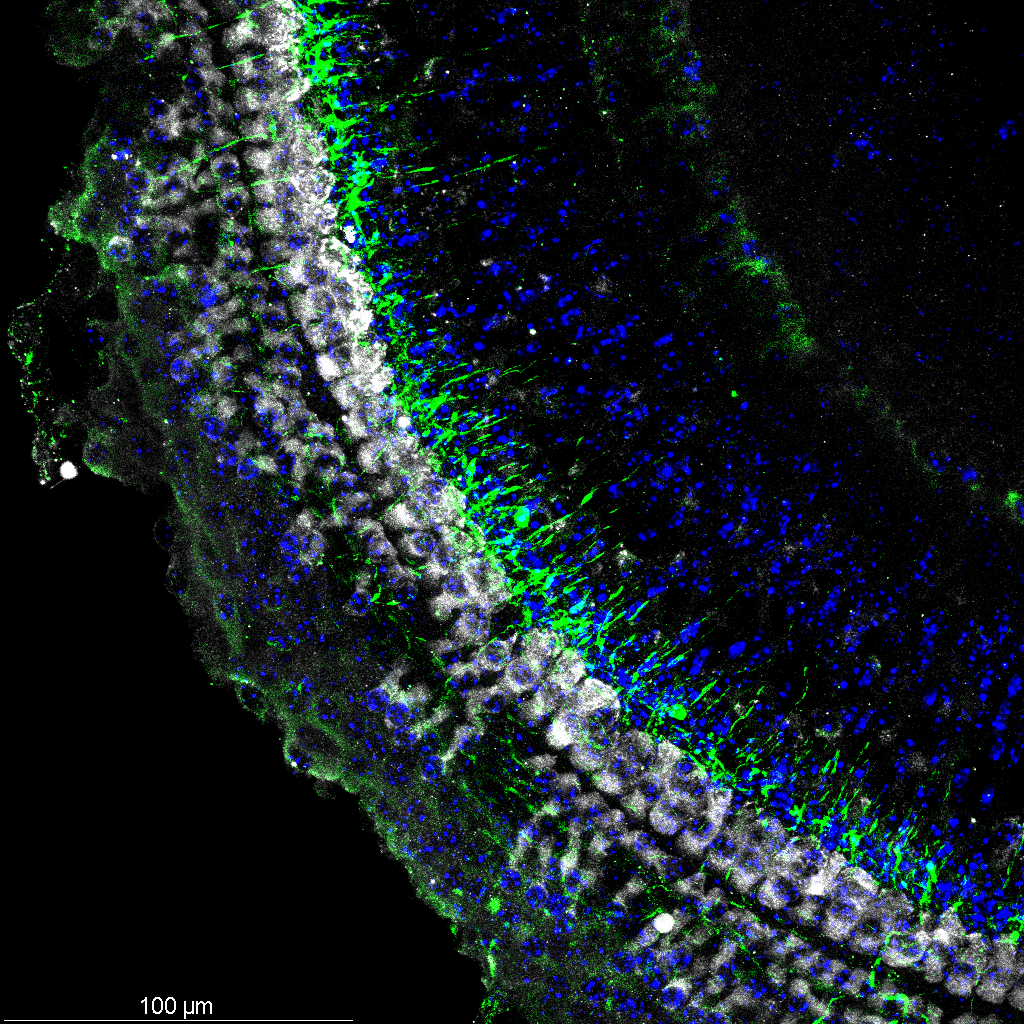


（2）Apex-12m_M


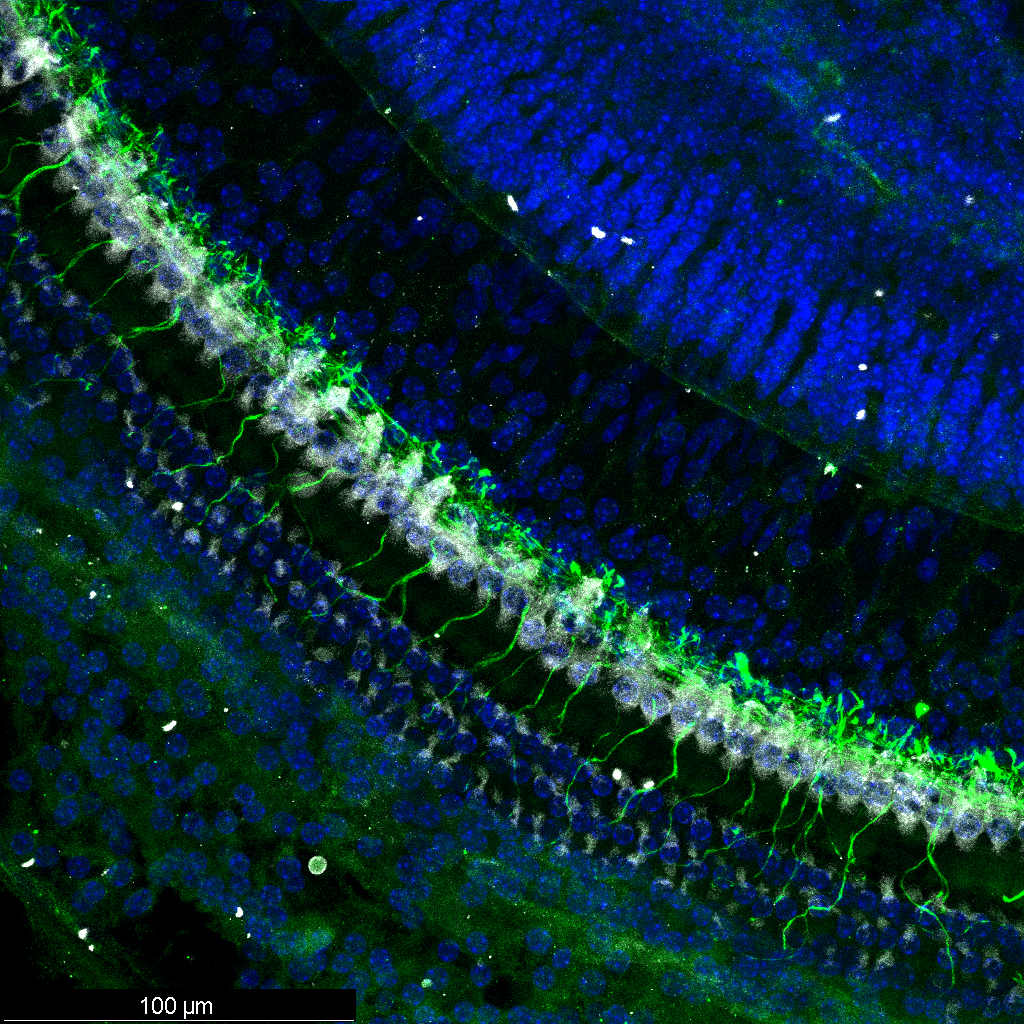


（3）Apex:6w


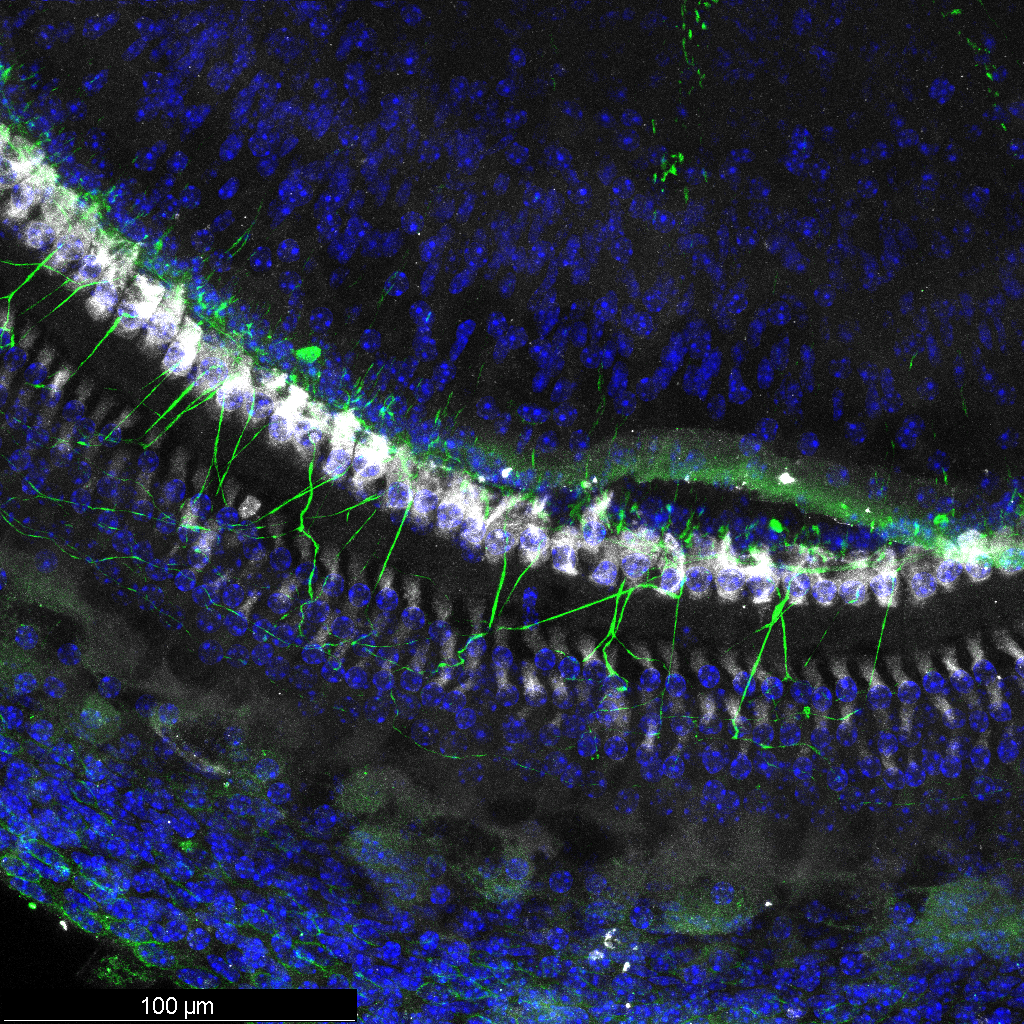


Figure 2D

1. Middle:12m_S


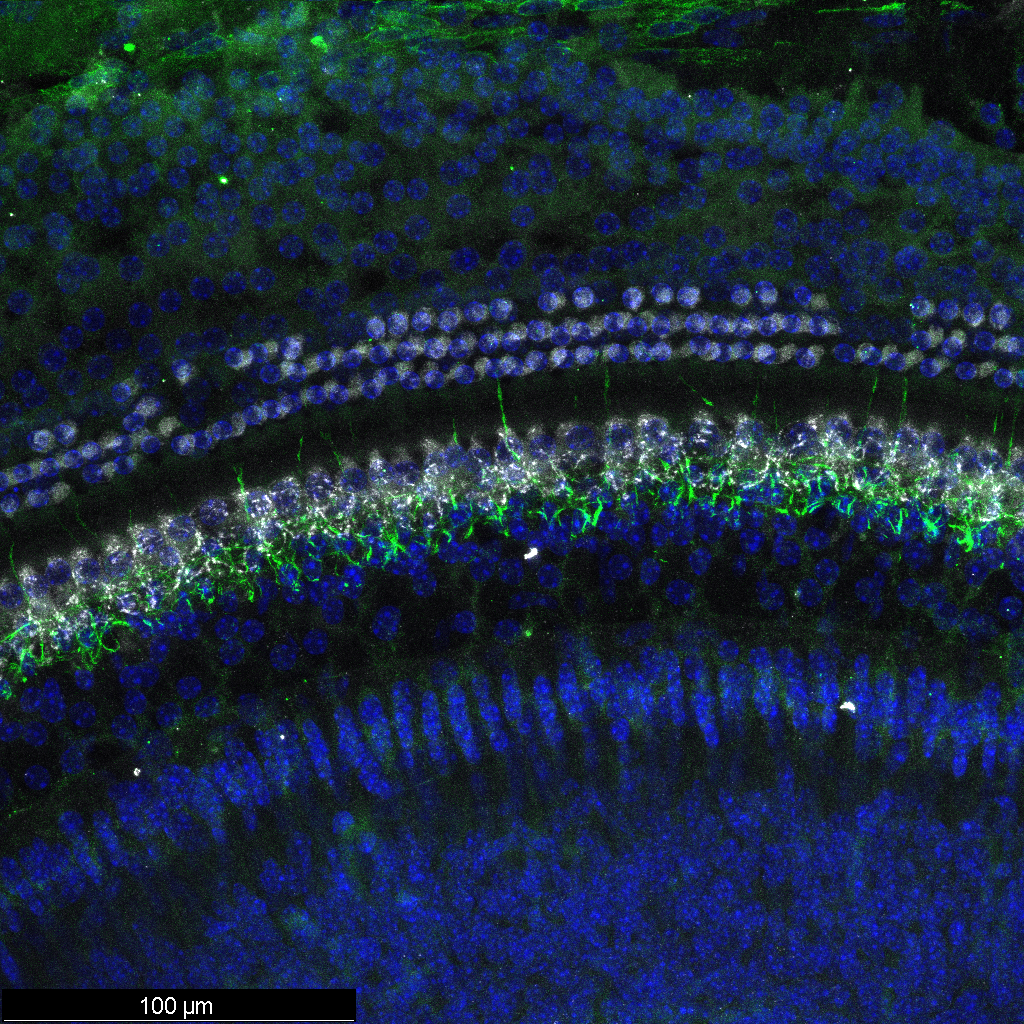


1. Middle:12m_M


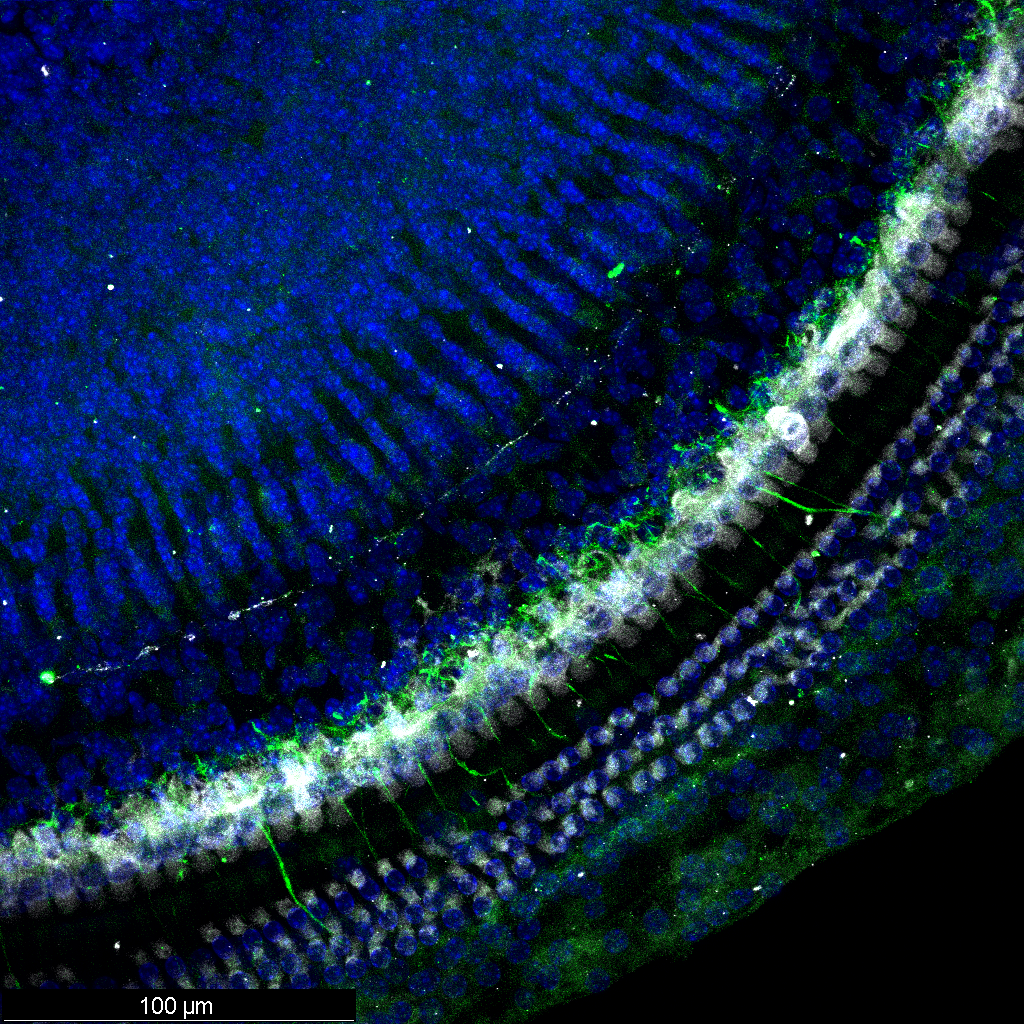


1. Middle:6w


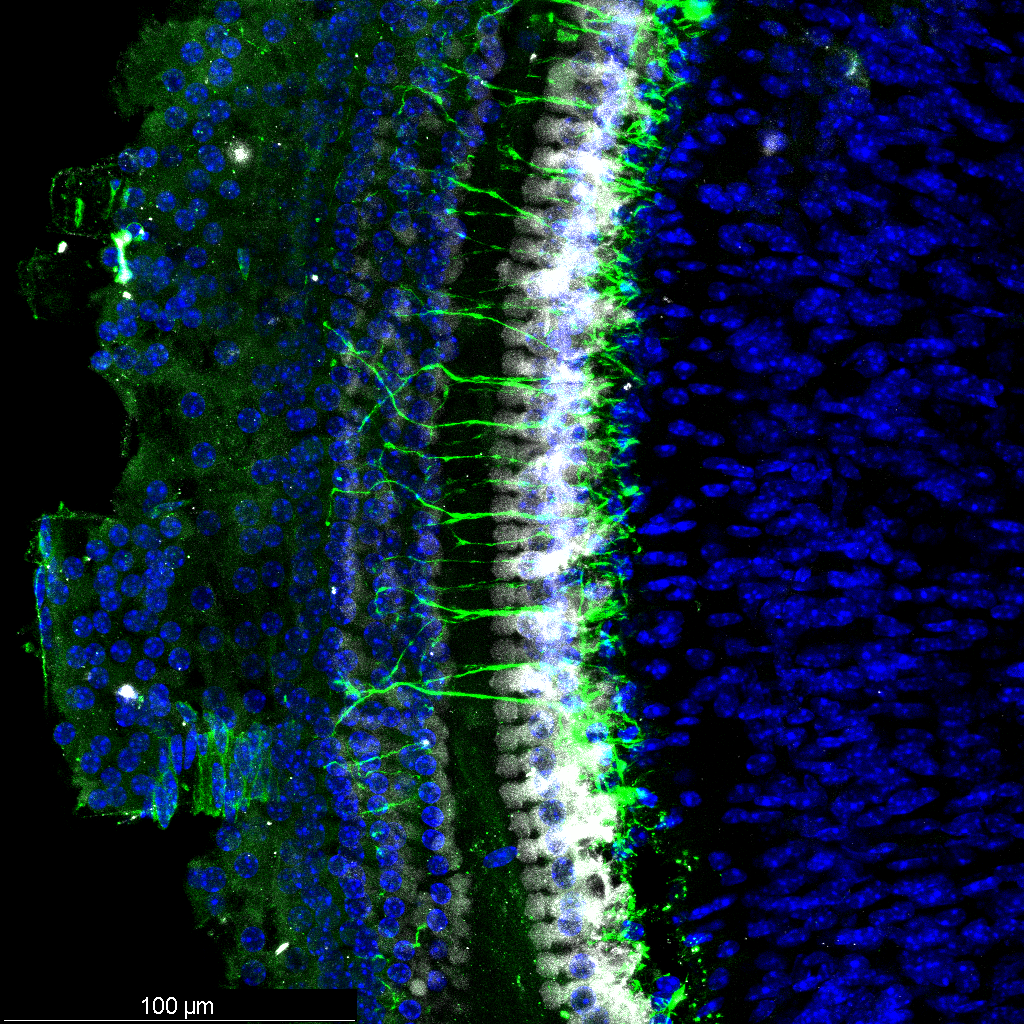


Figure 2E

（1）Base:12m_S


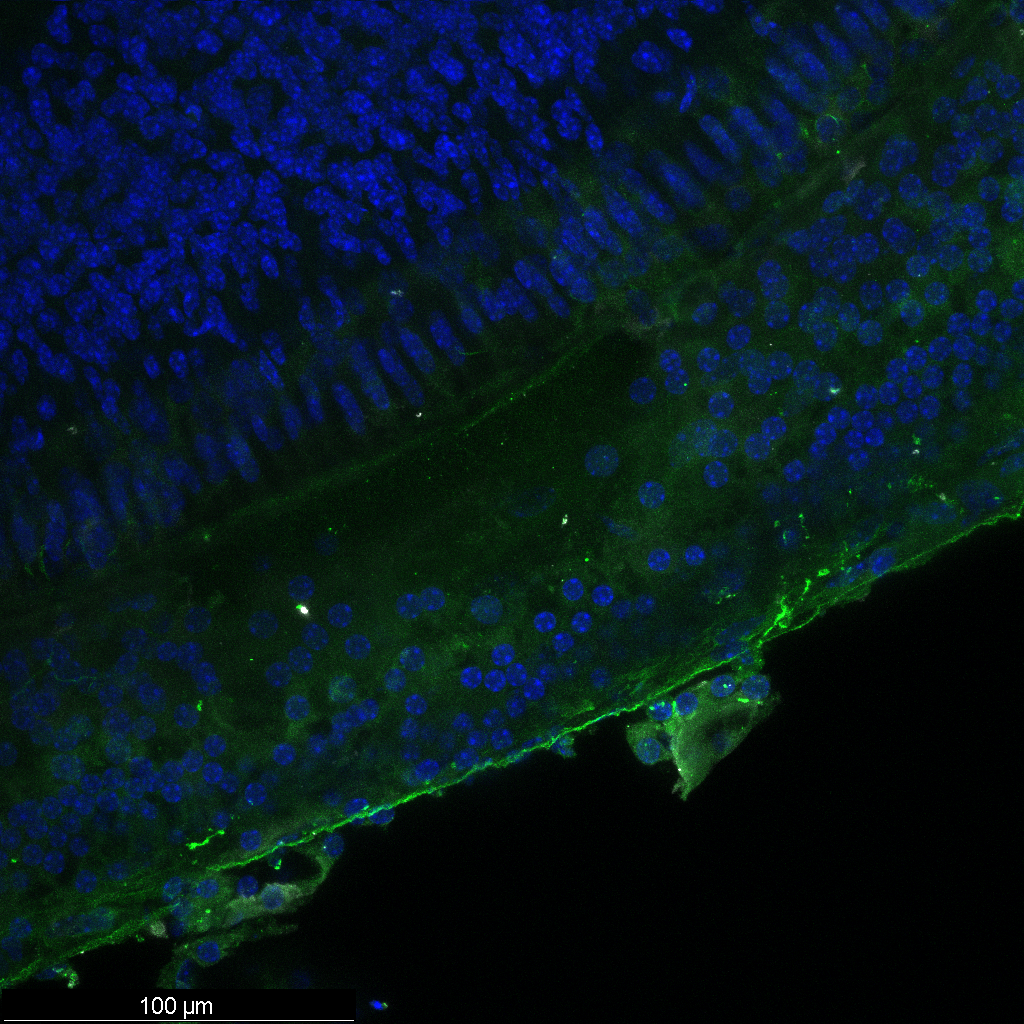


（2）Base:12m_M


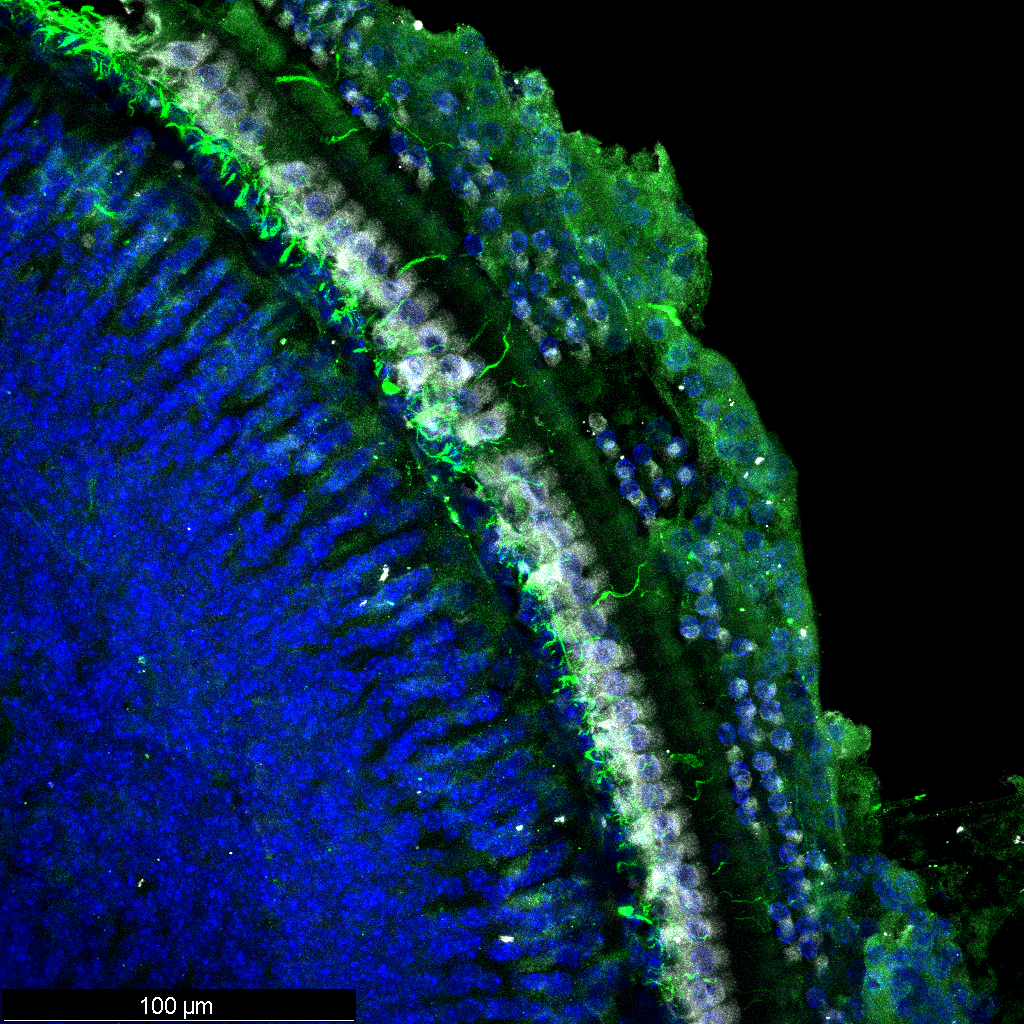


（3）Base:6w


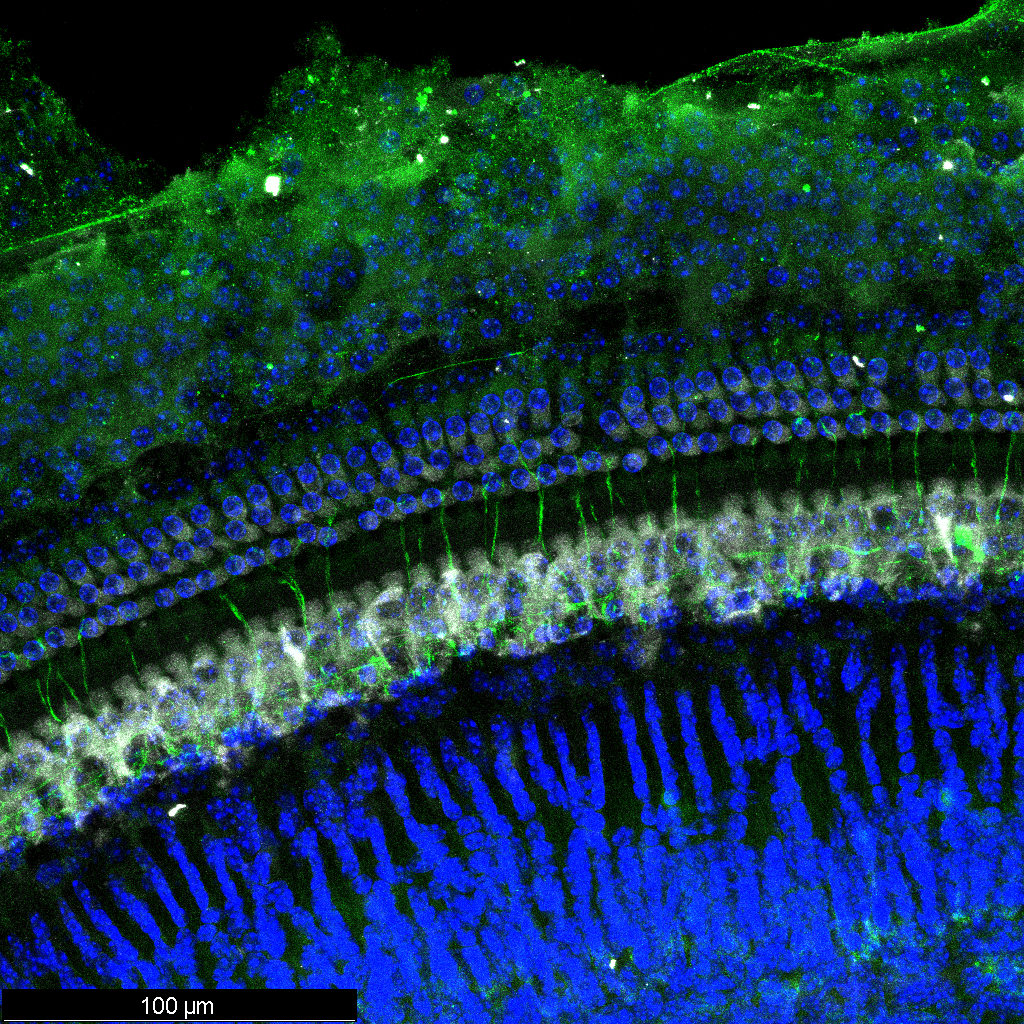


Figure G

（1）12m_S: Apex


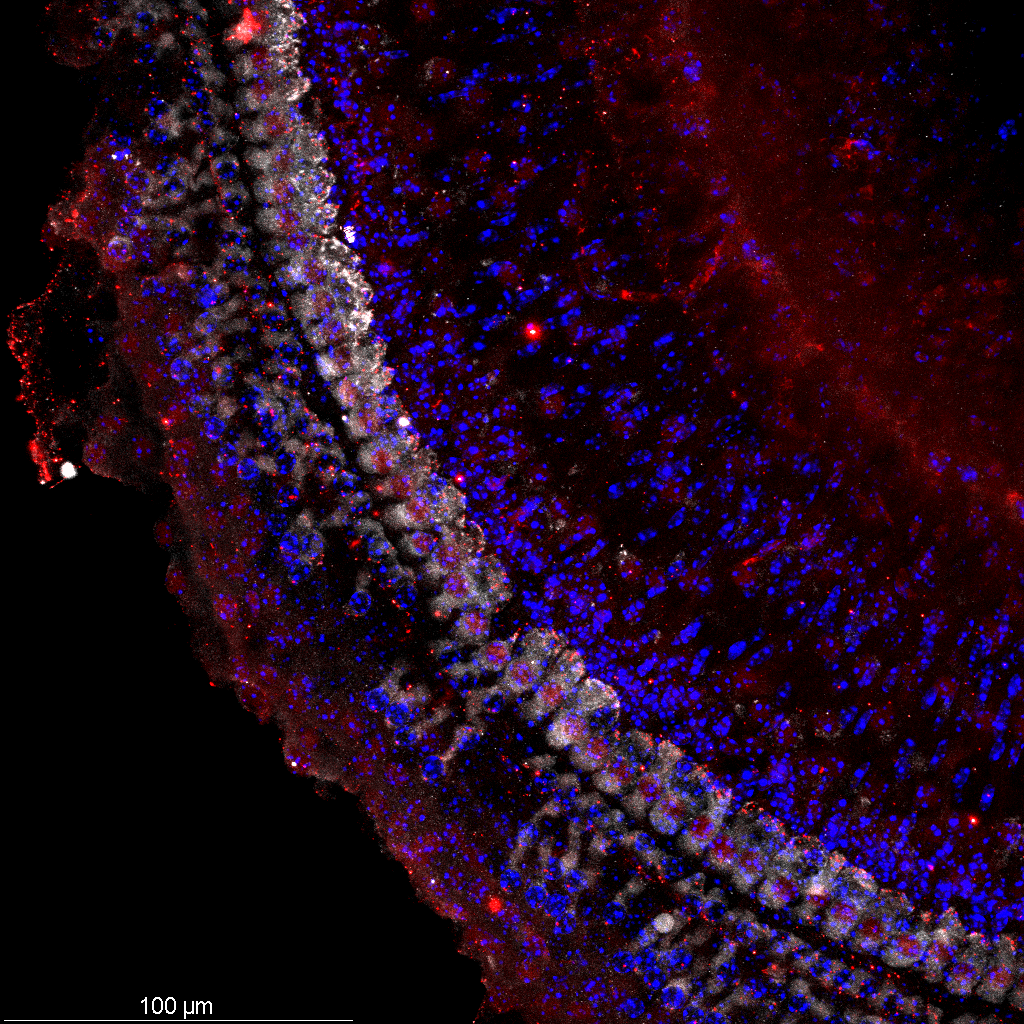


1. 12m_S: Middle


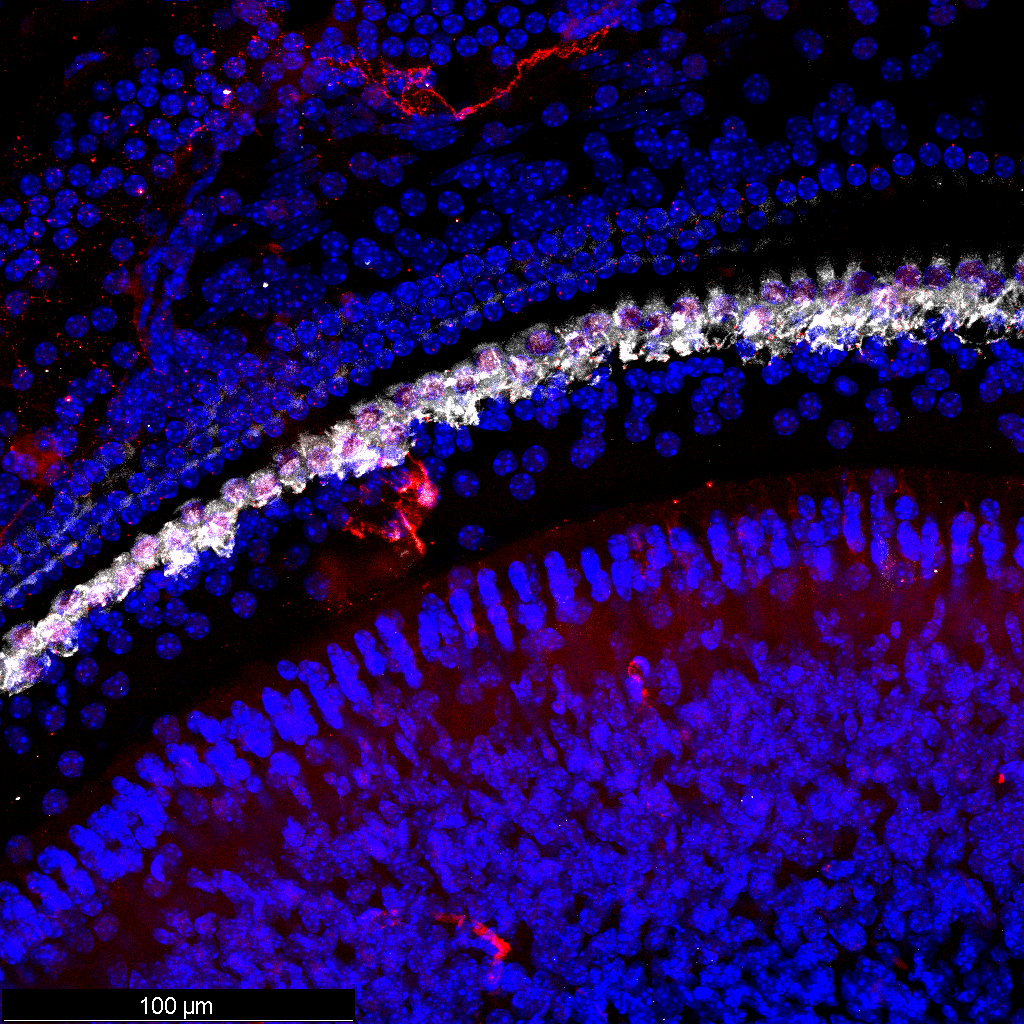


1. 12m_S: Base


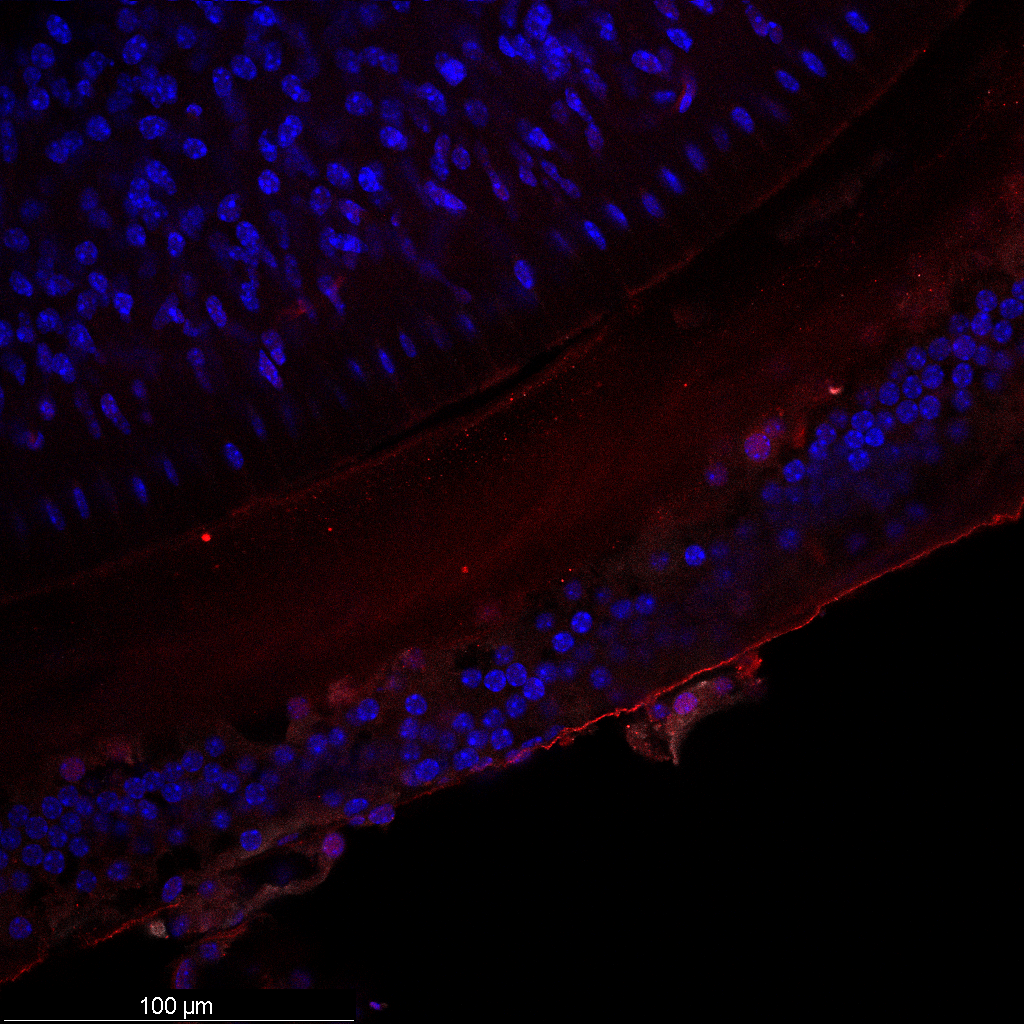


1. 12m_M: Apex


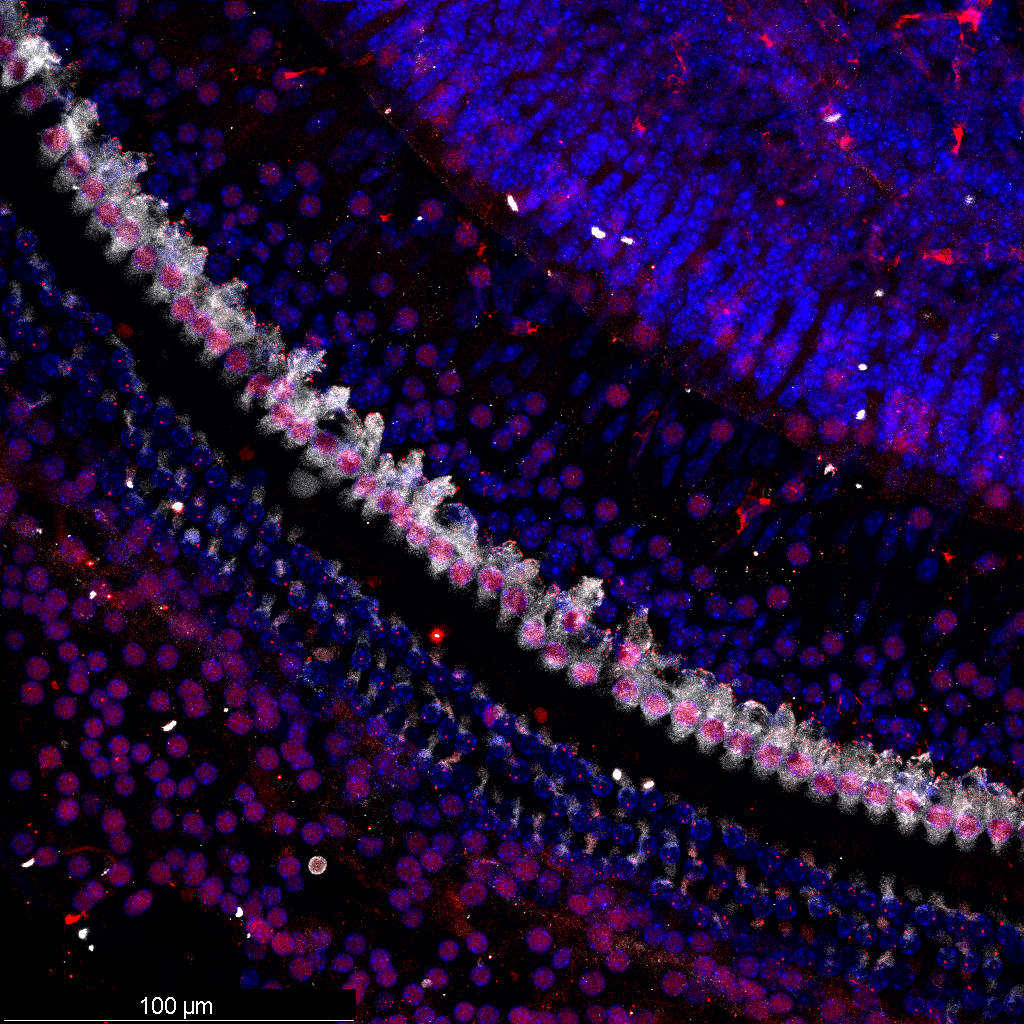


1. 12m_M: Middle


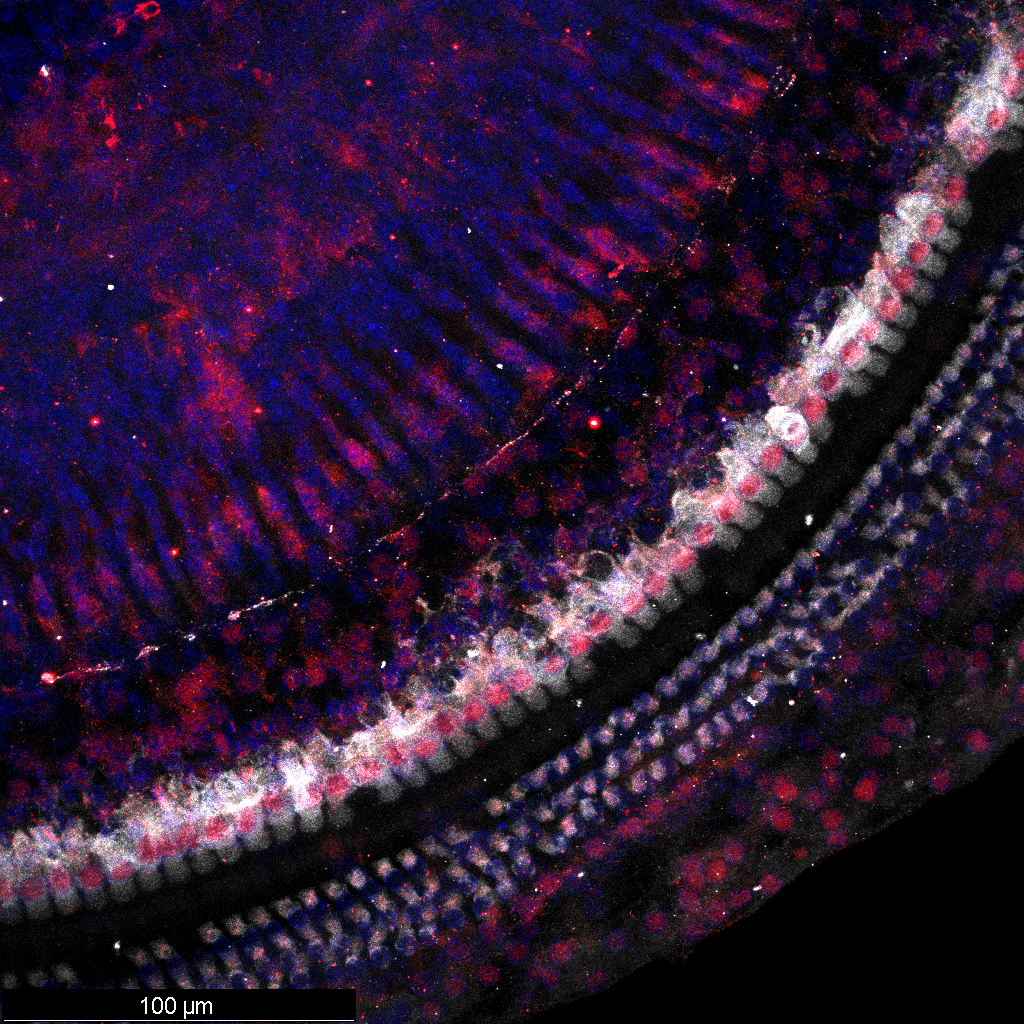


1. 12m_M: Base


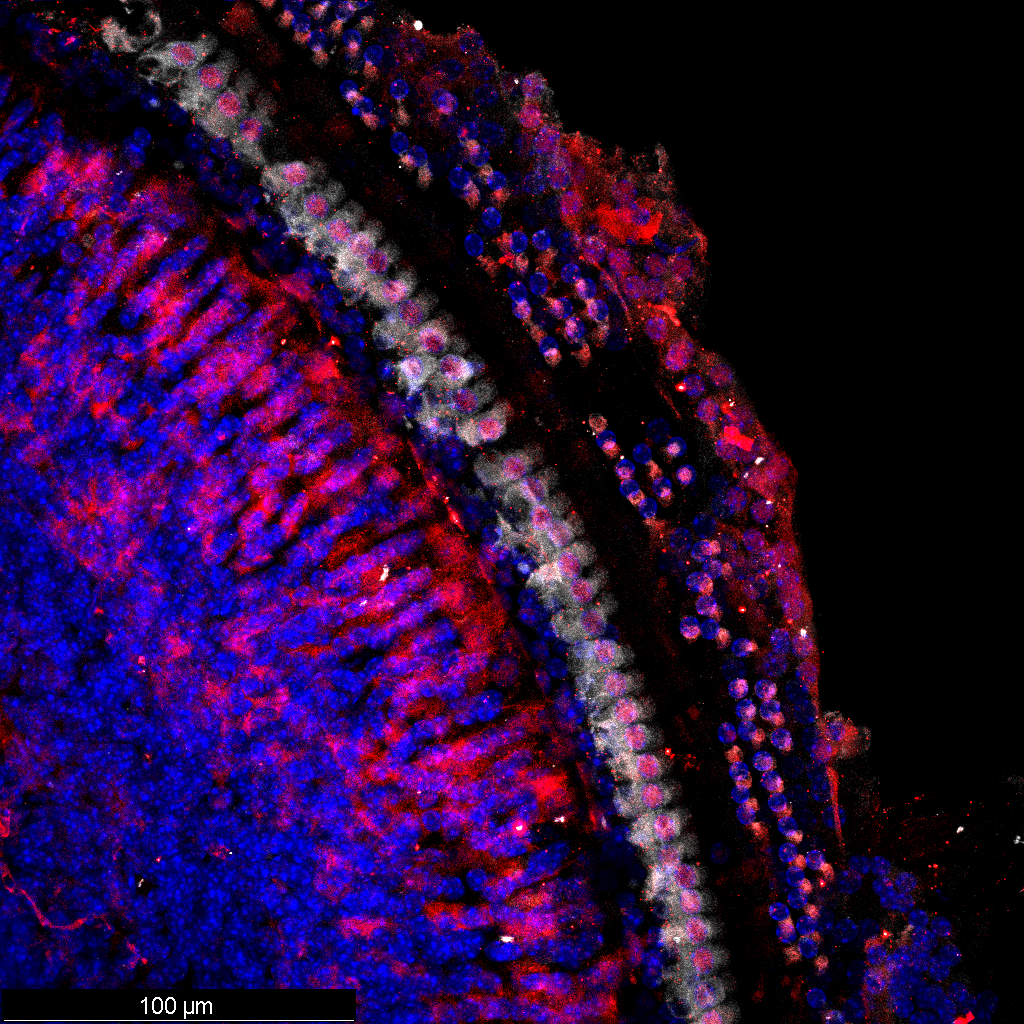


1. 6w: Apex


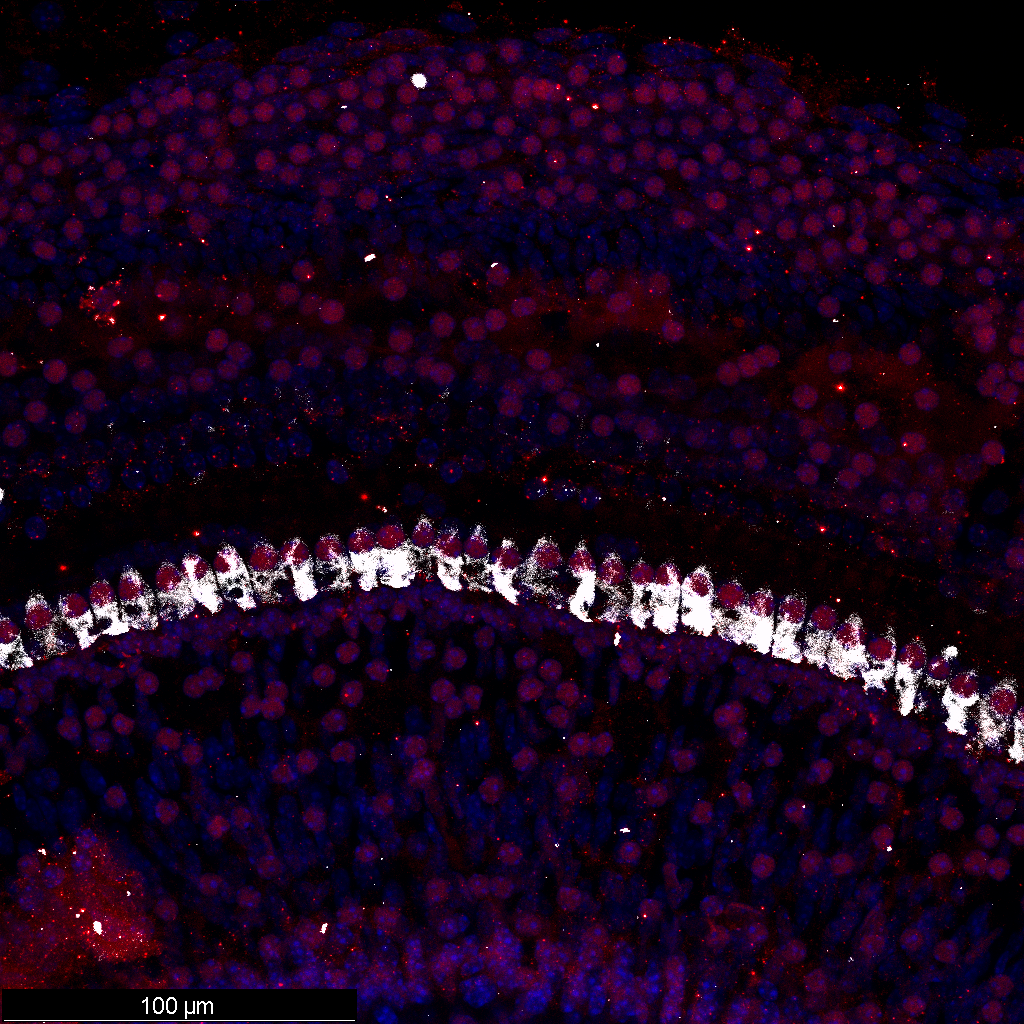


1. 6w: Middle


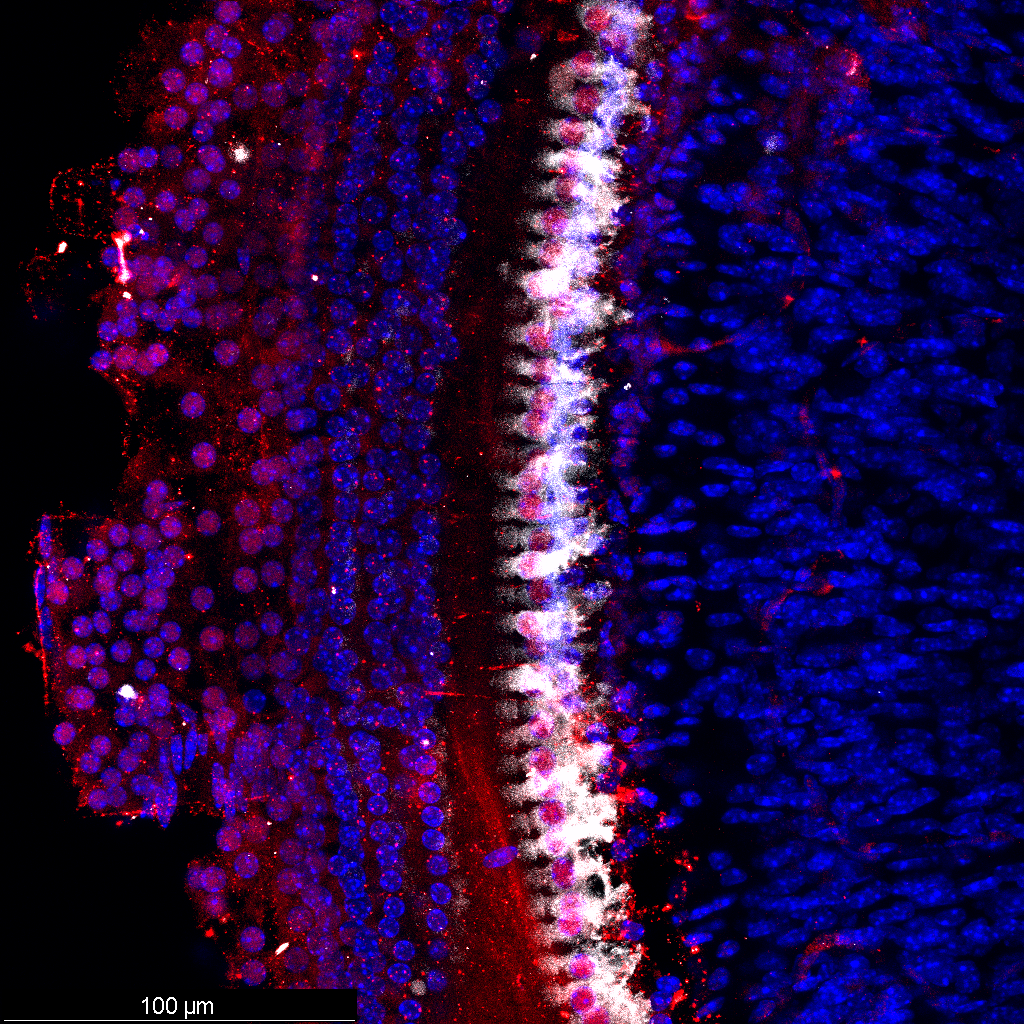


1. 6w: Middle


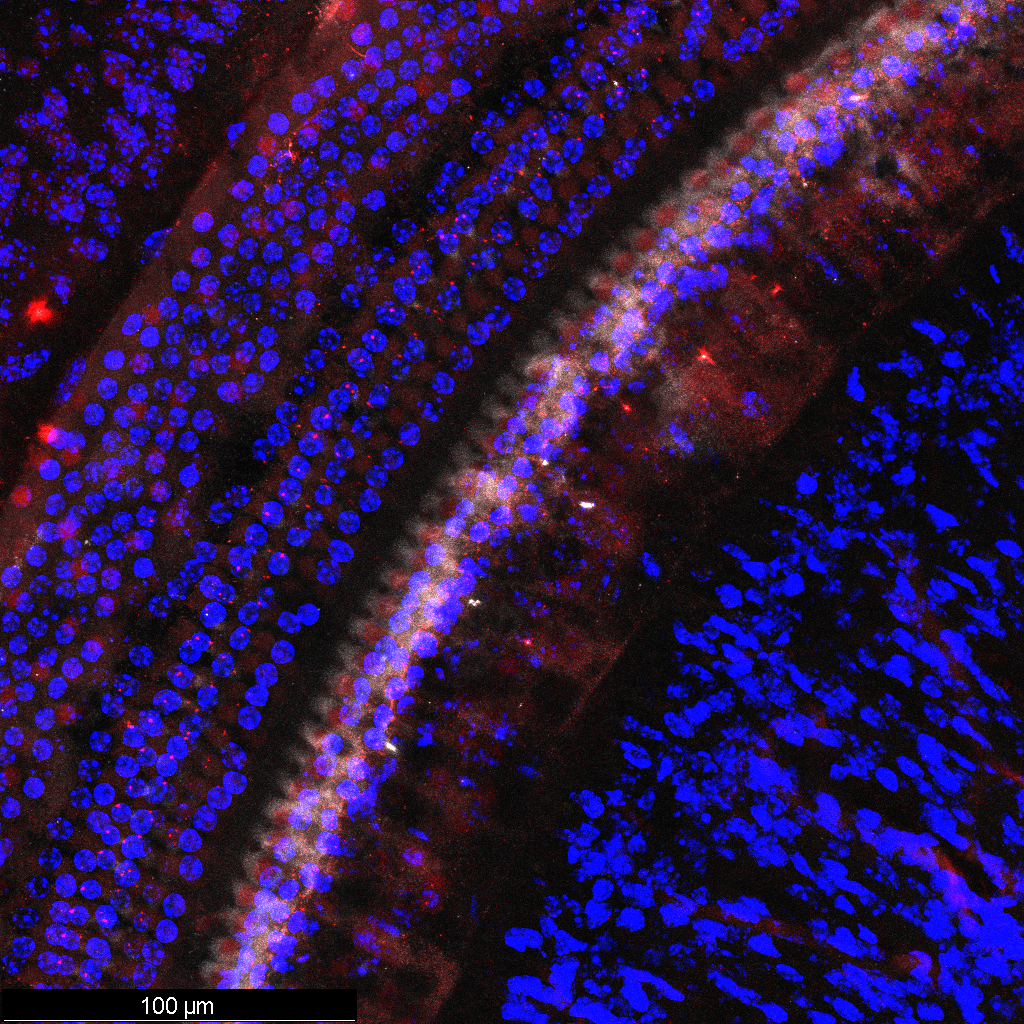


Figure 3D

1. Control-Apex


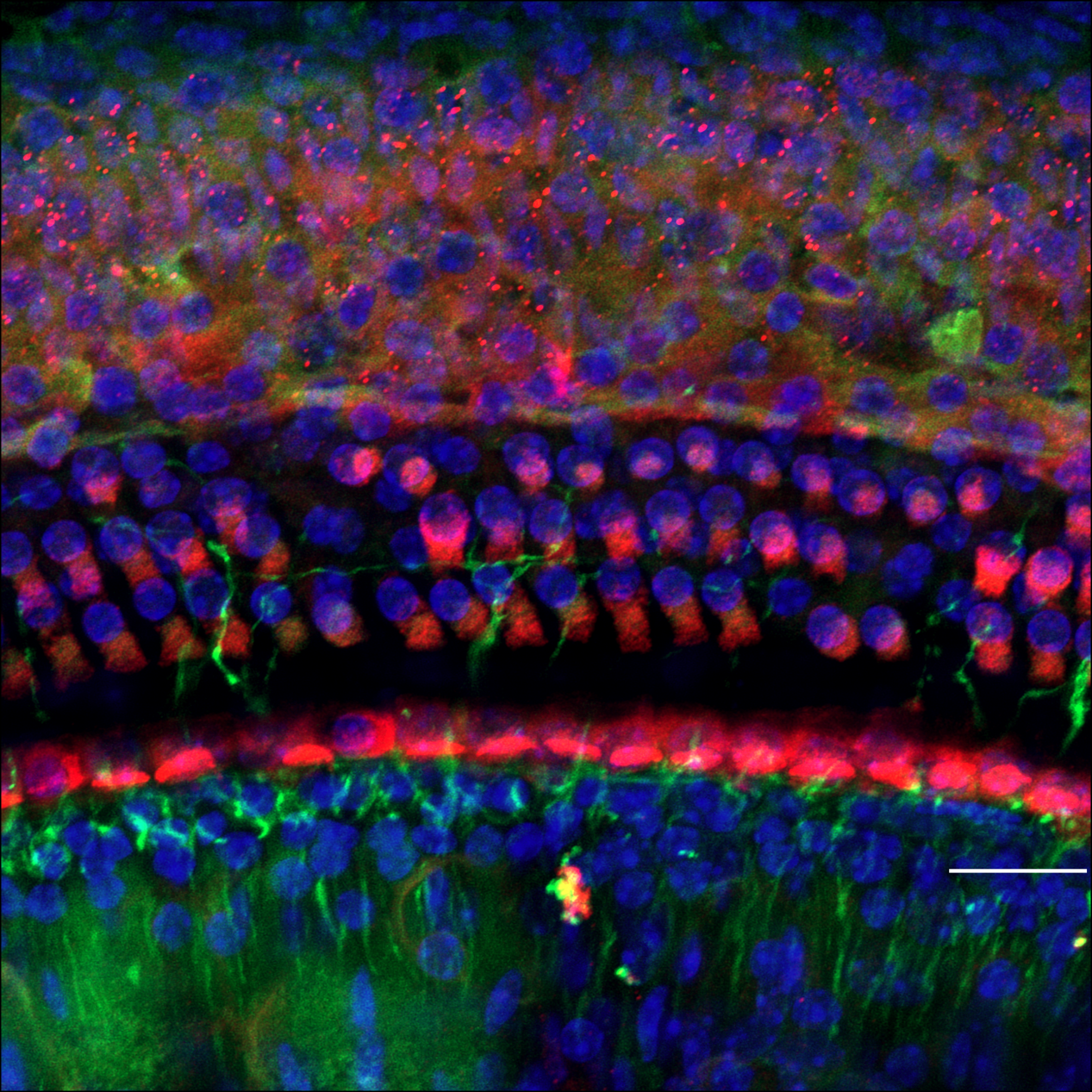


1. Control-Middle


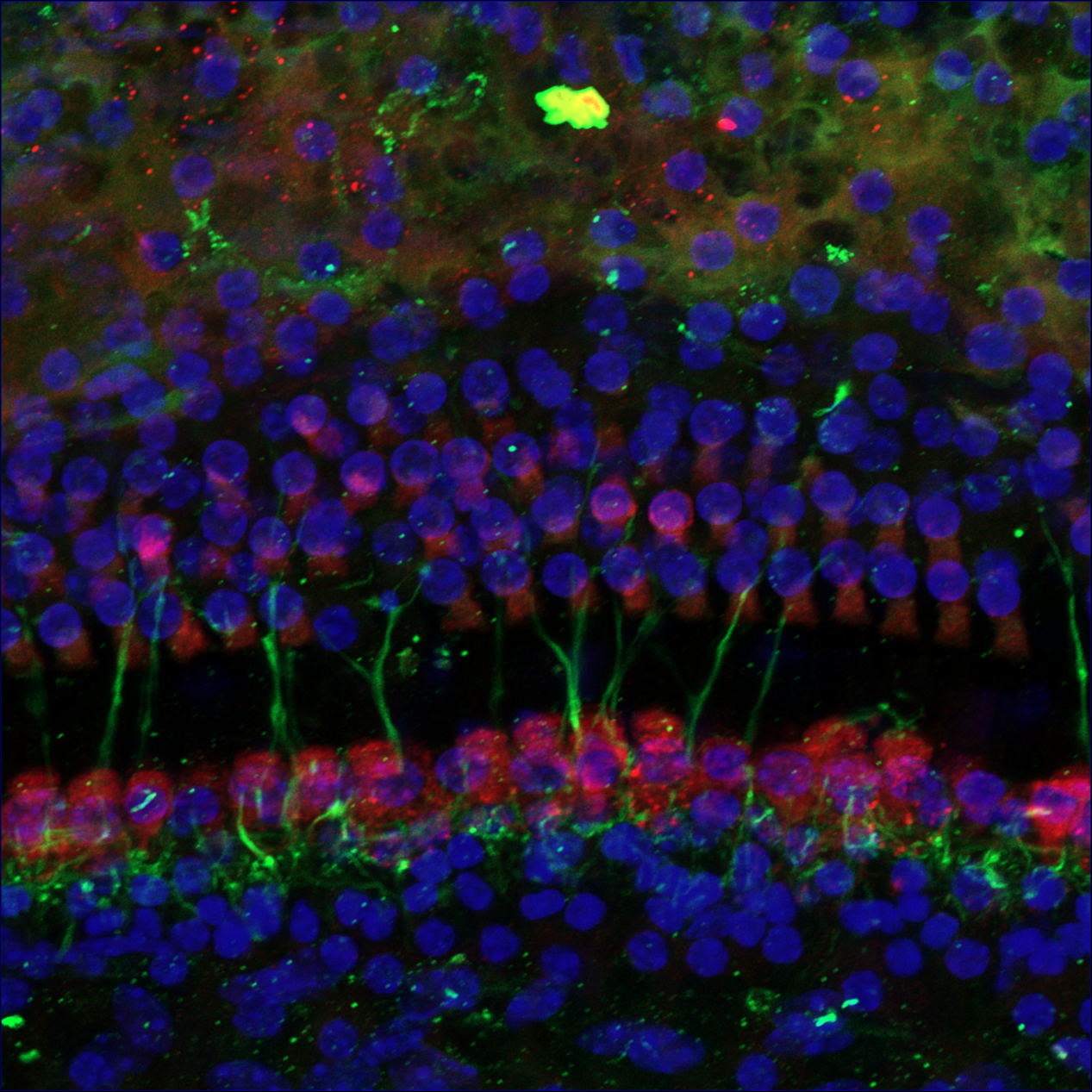


1. Control-Base


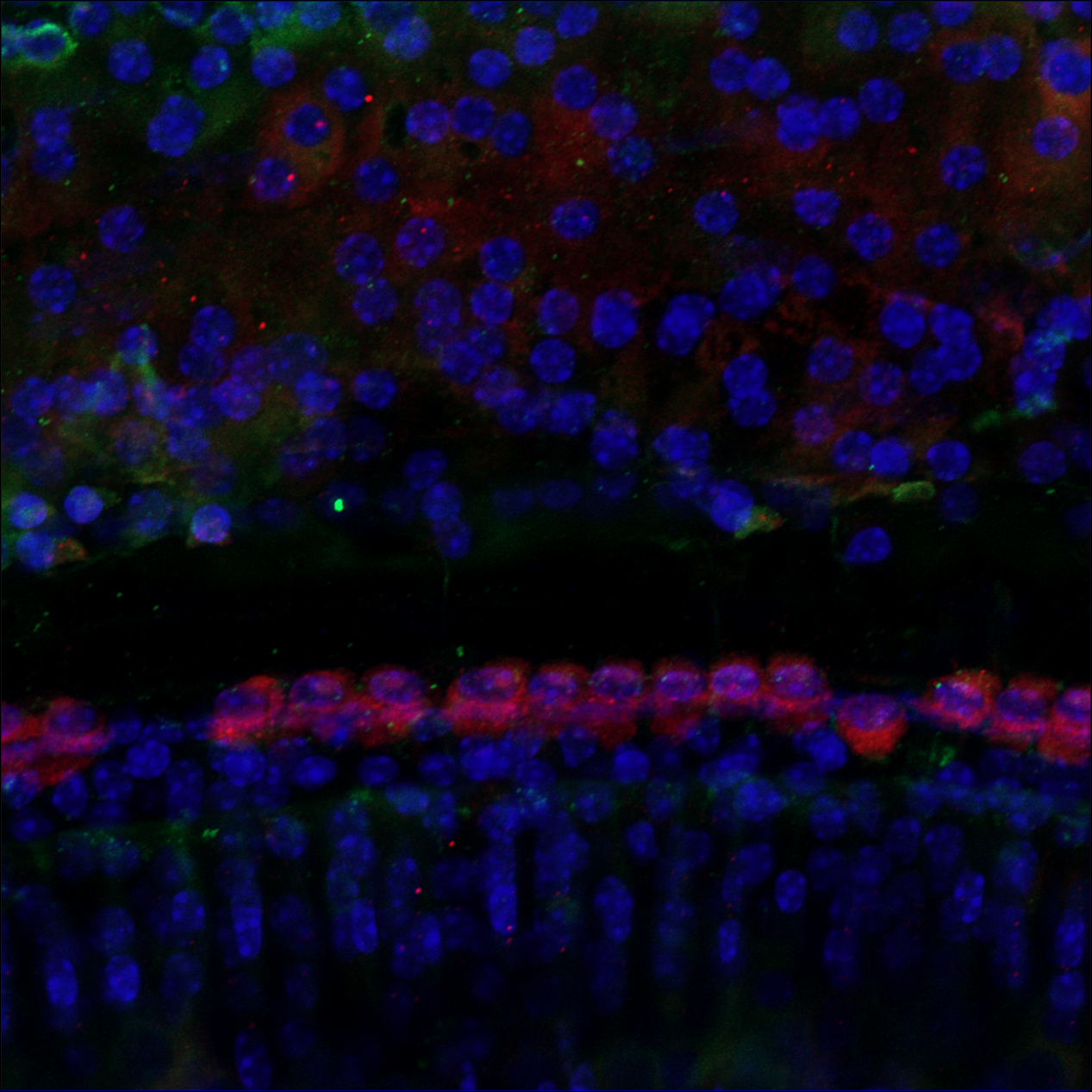


1. FMT-6w-Apex


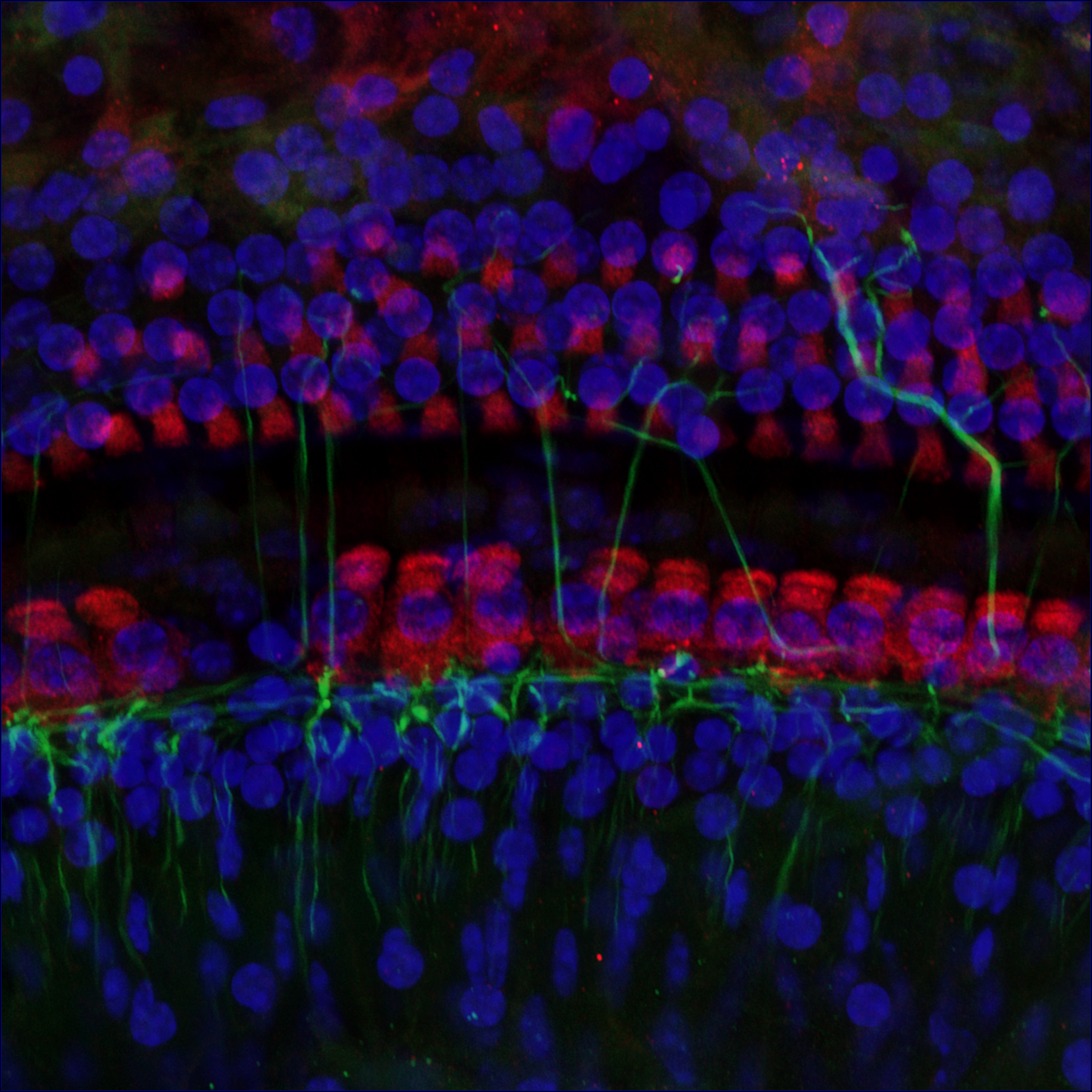


1. FMT-6w-Middle


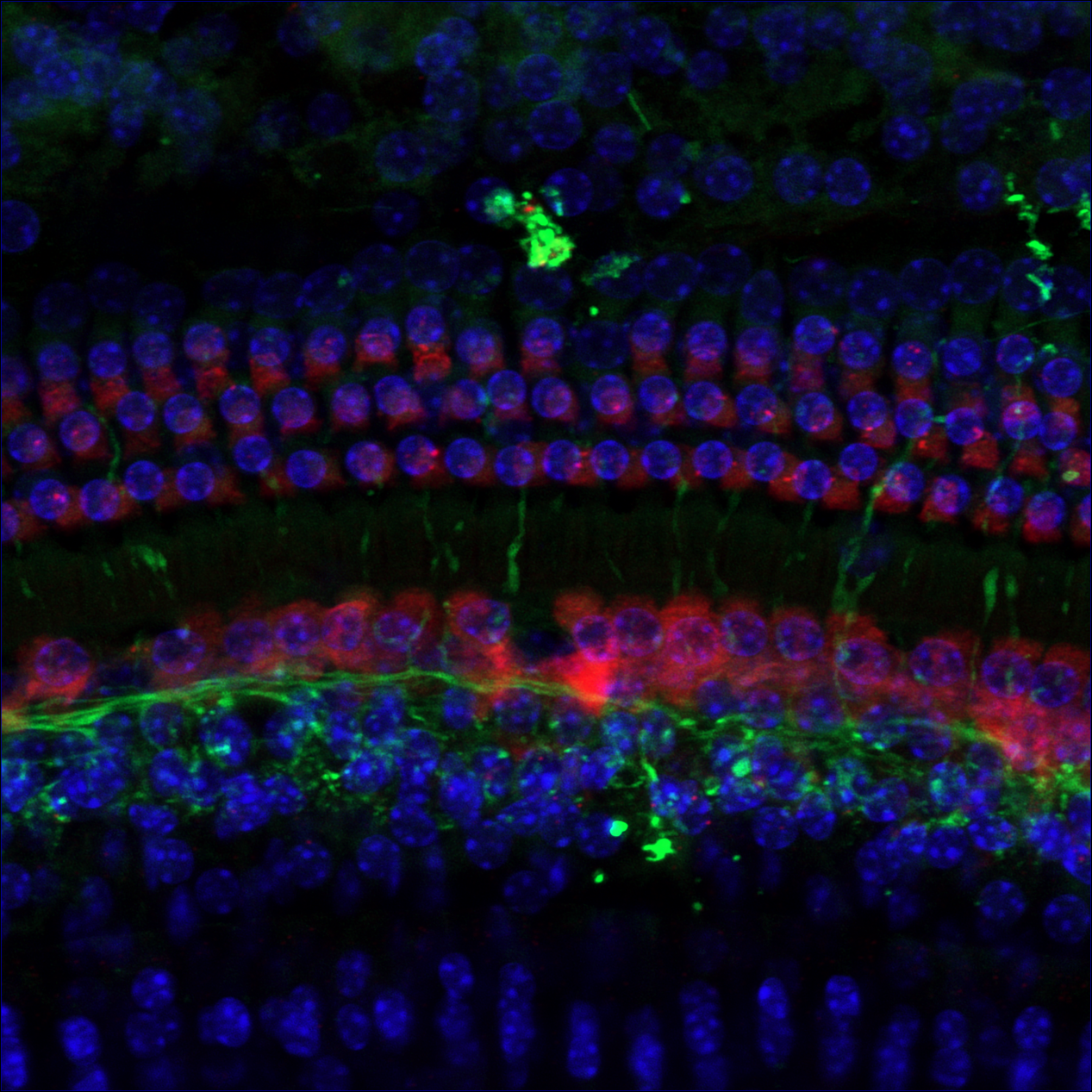


1. FMT-6w-Base


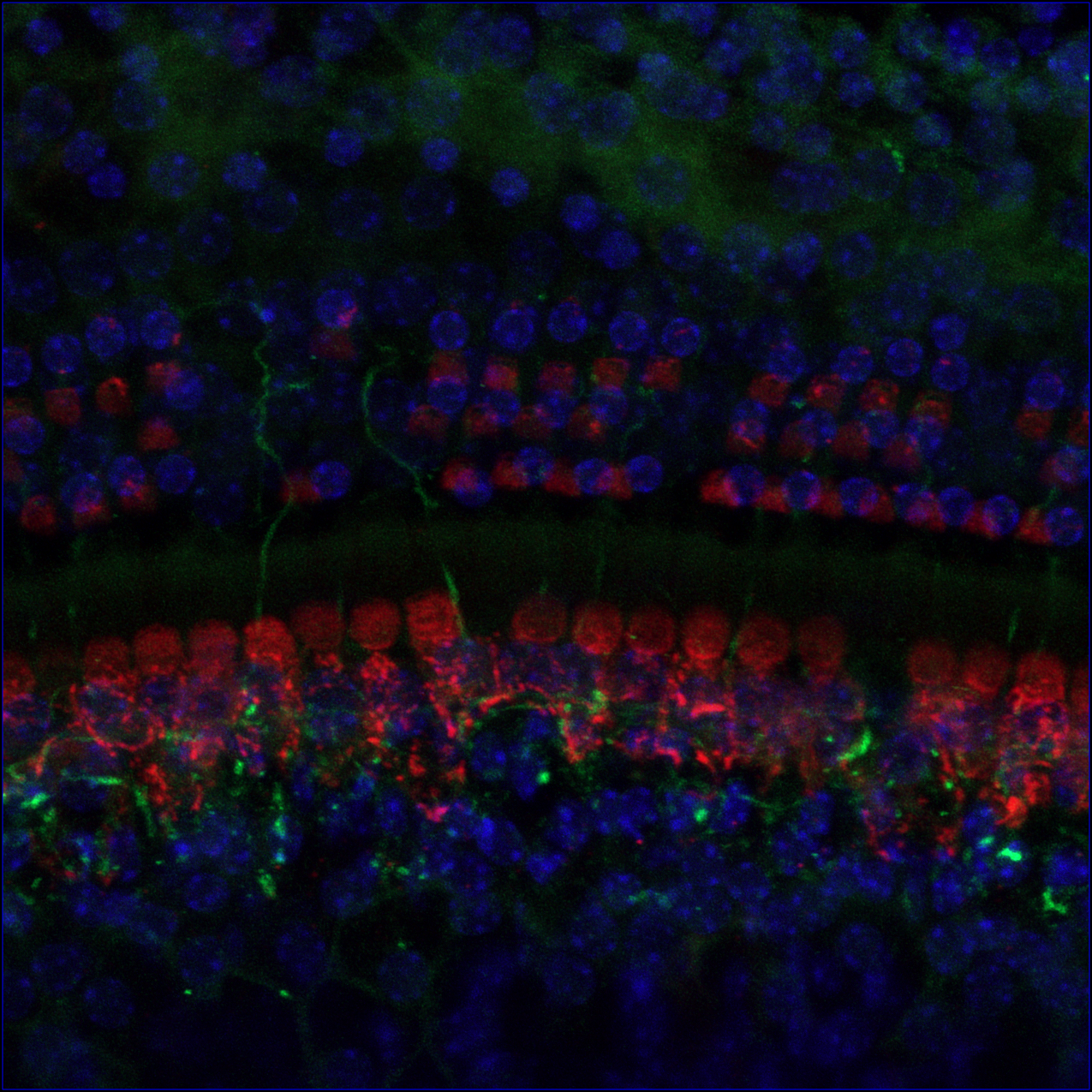


1. FMT-12m_S-Apex


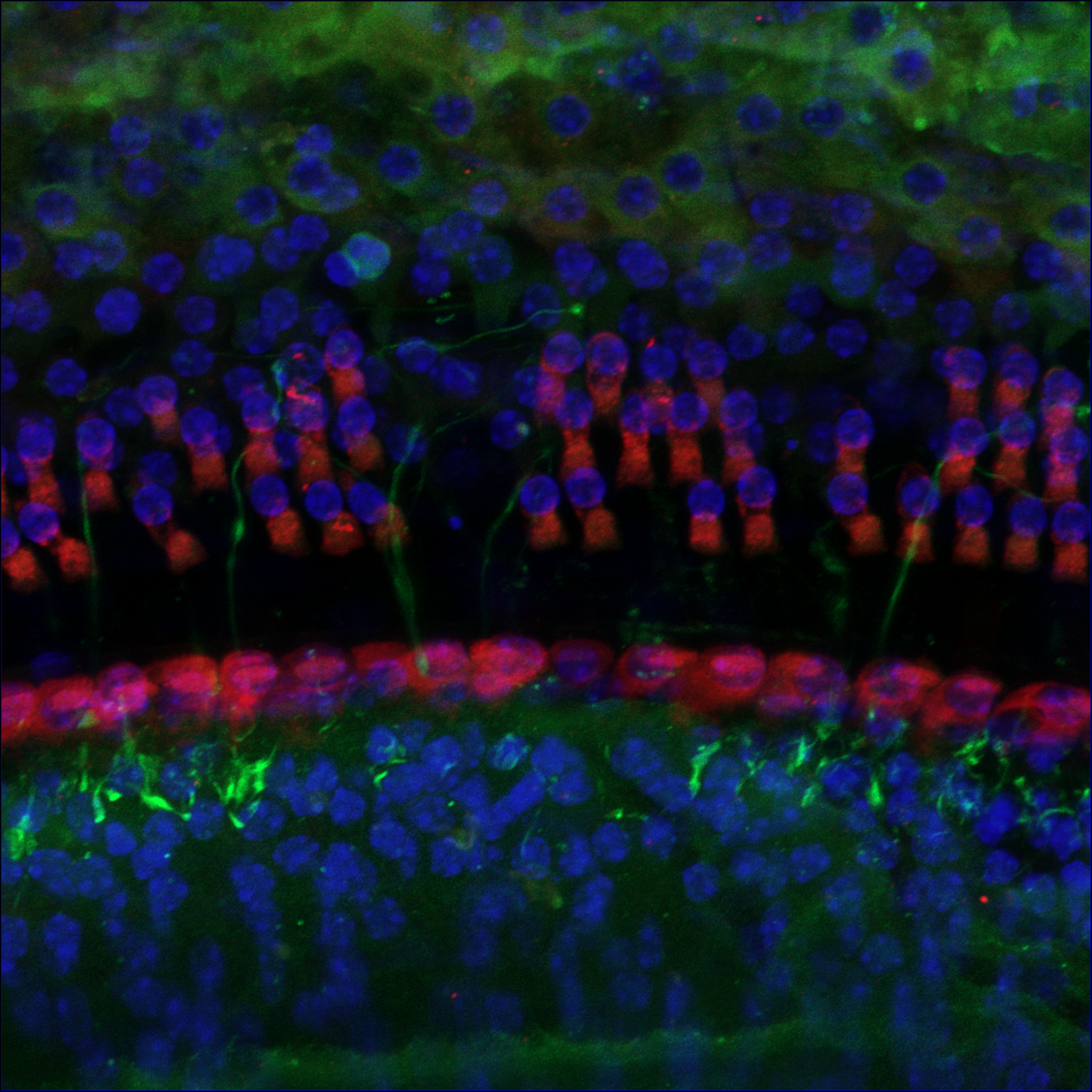


1. FMT-12m_S-Middle


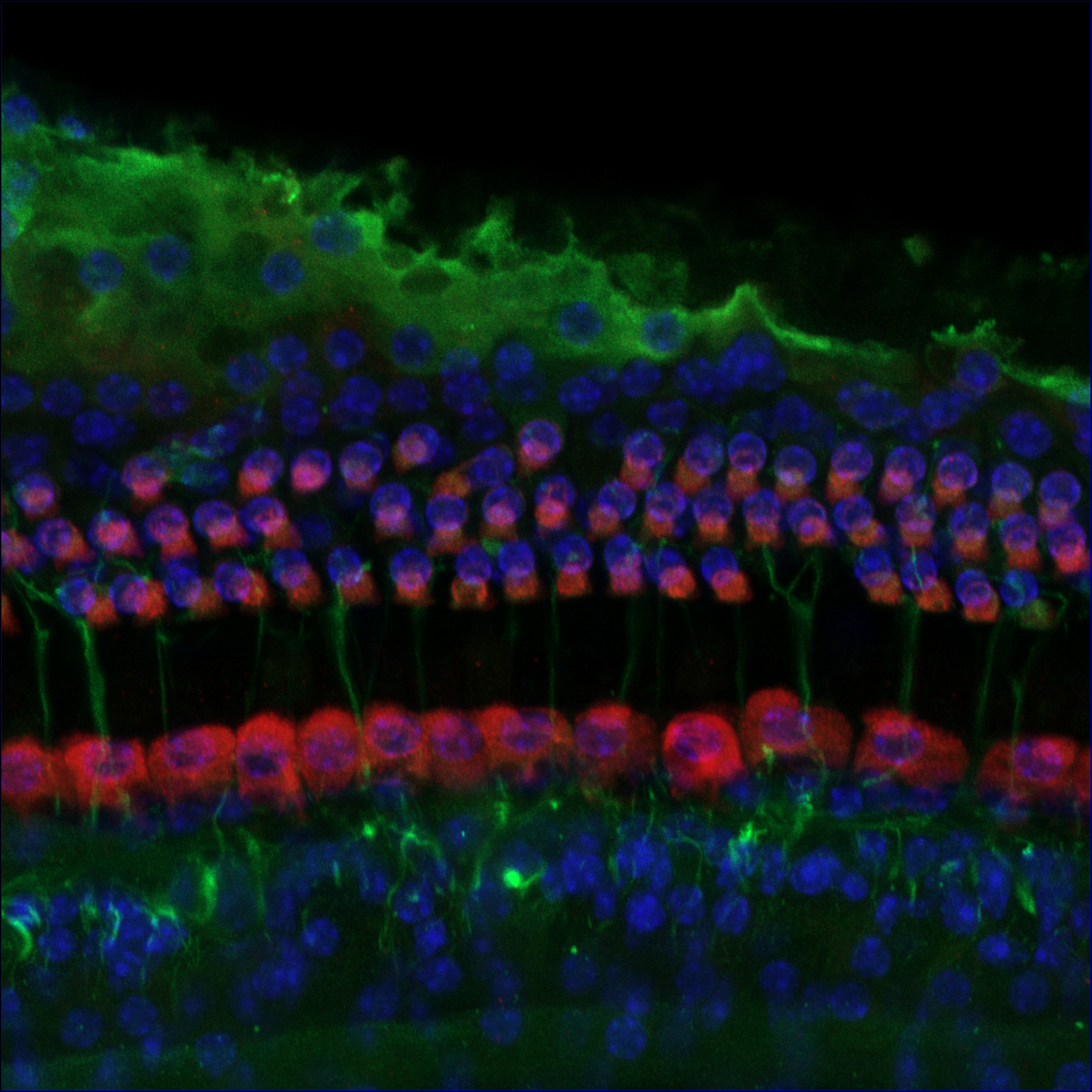


1. FMT-12m_S-Base


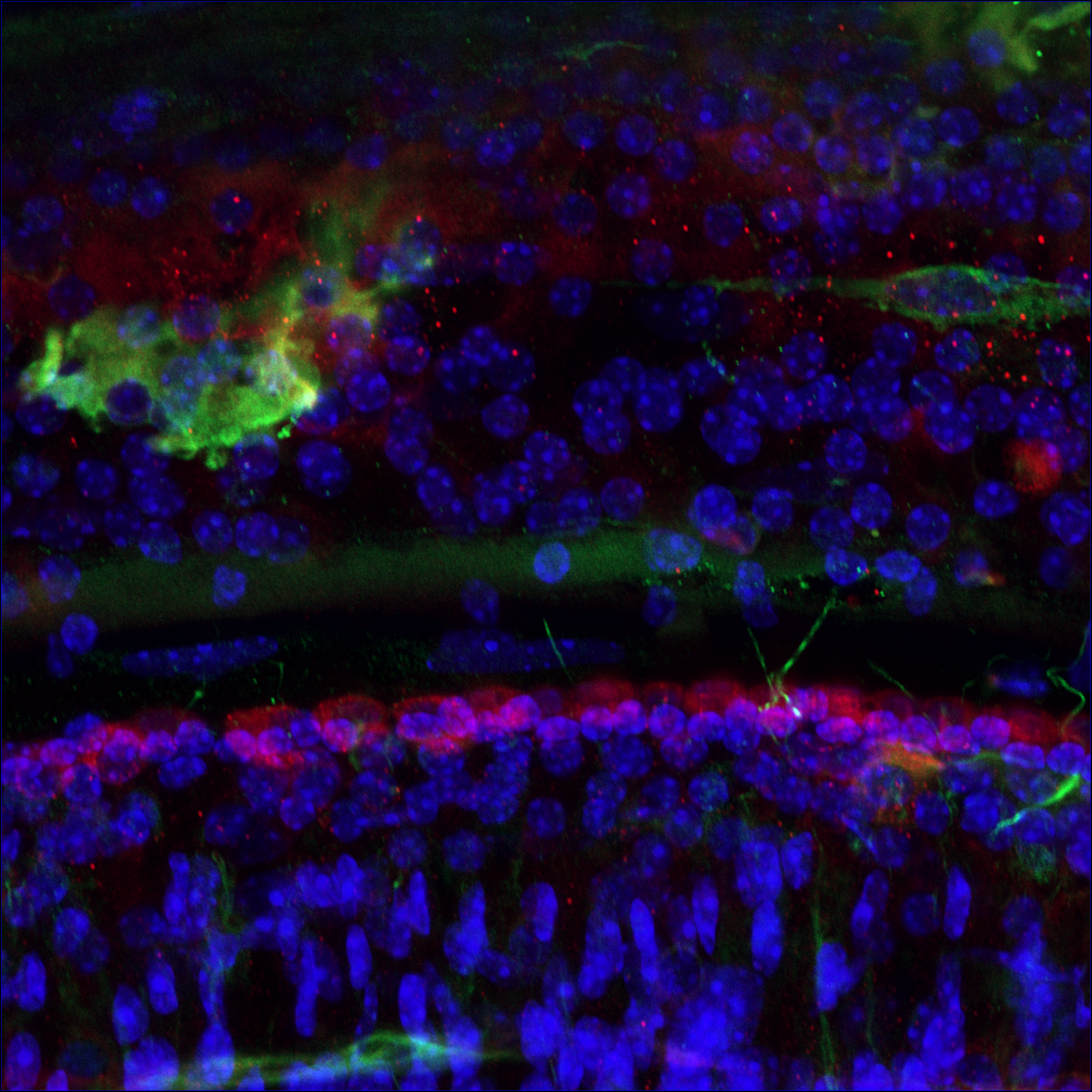


Figure 7C

1. Control


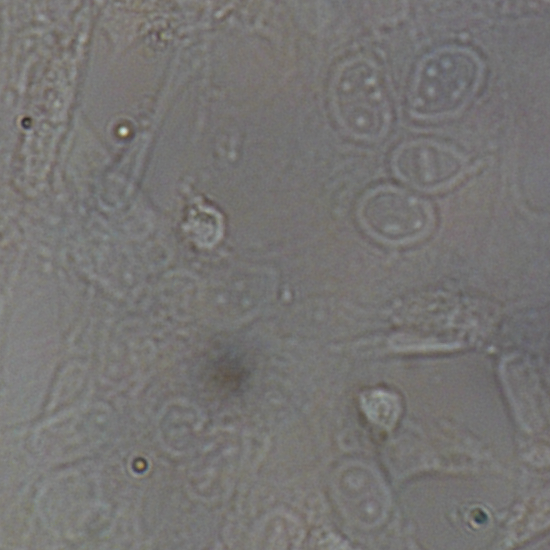


1. D-gal


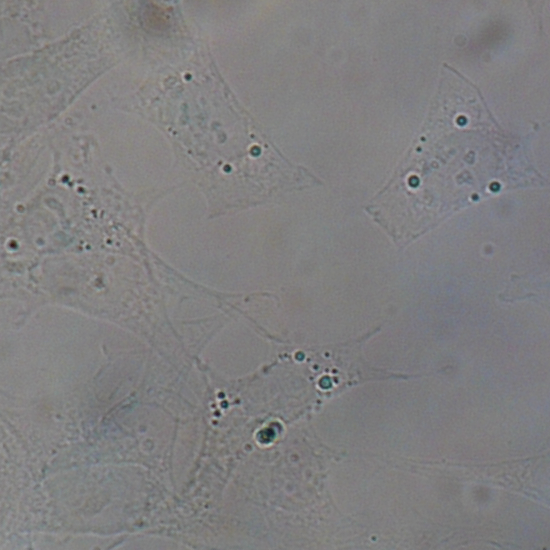


1. D-gal+5-HTP


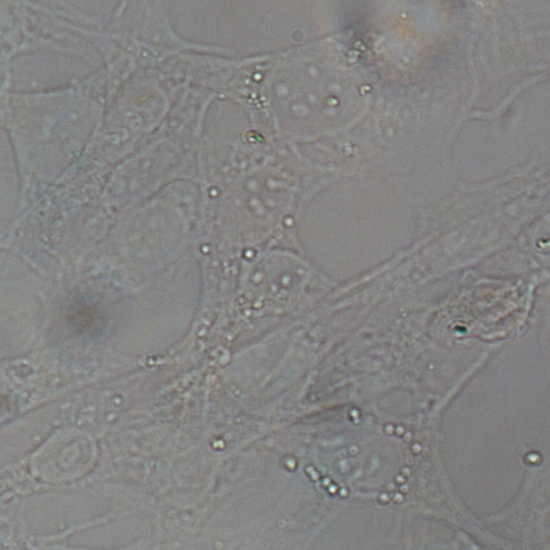


Figure 7D

1. Cellrox-Control


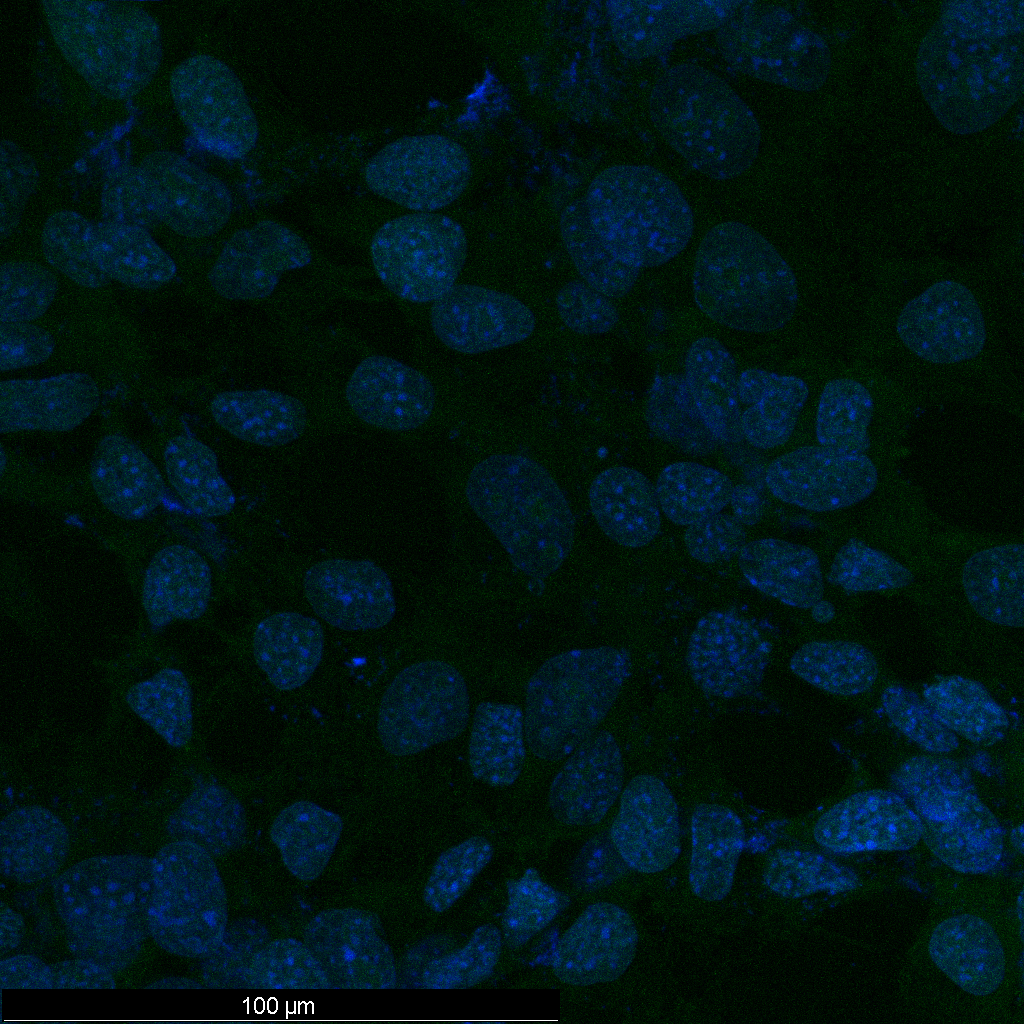


1. Cellrox-D-gal


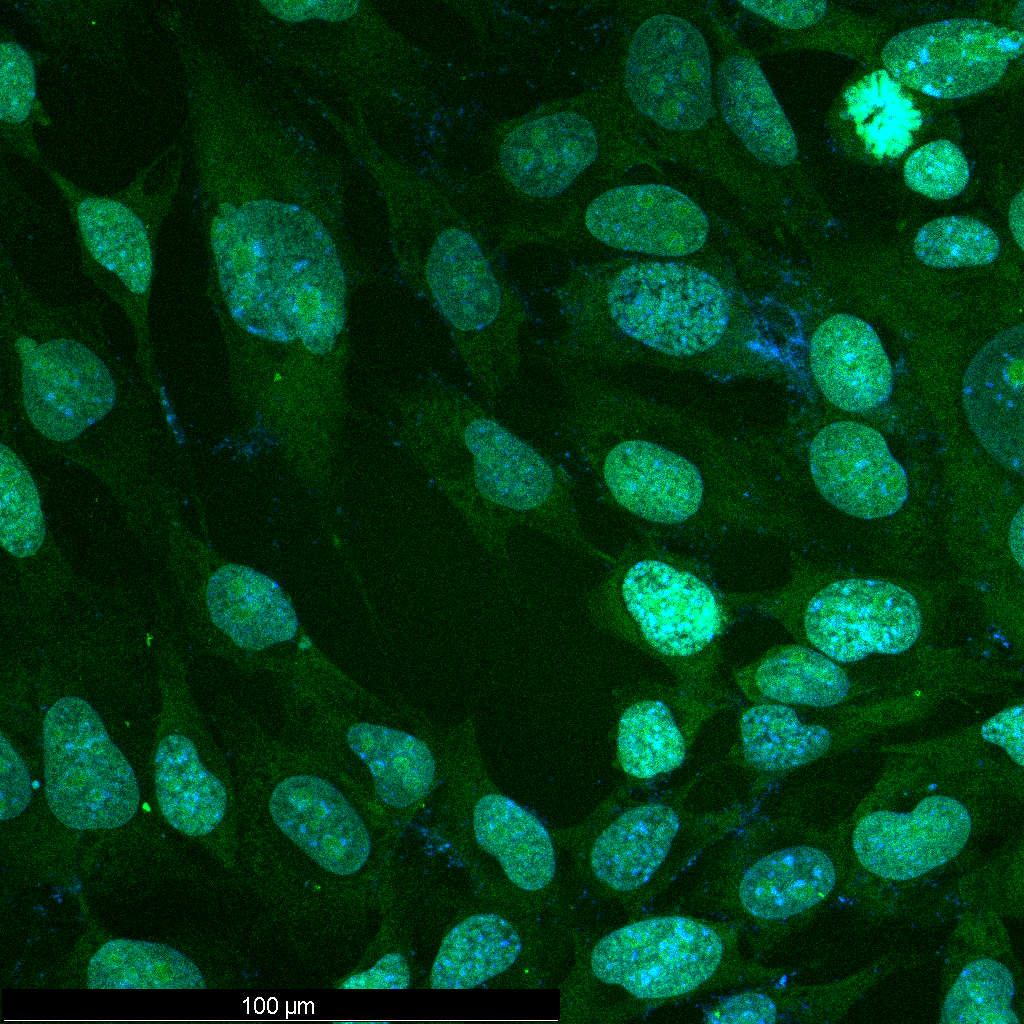


1. Cellrox-D-gal+5-HTP


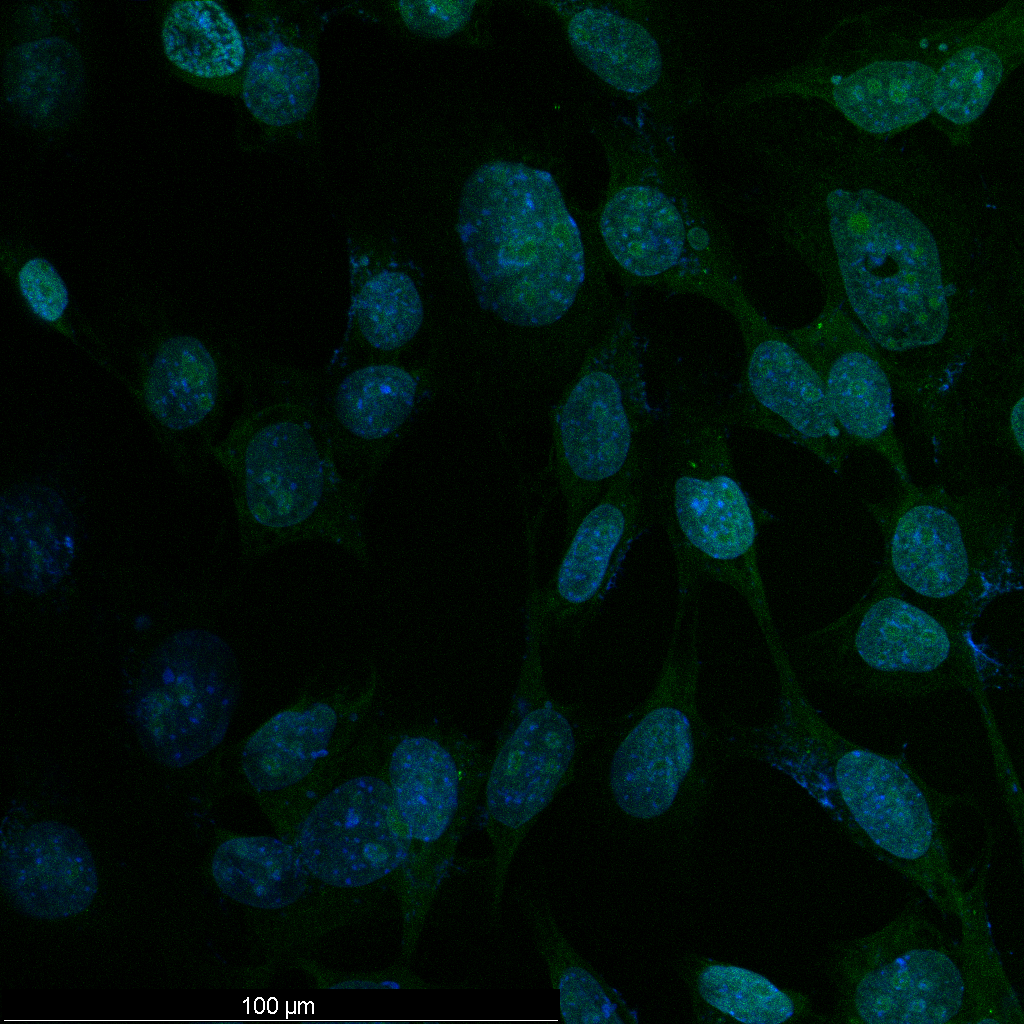


1. Mitosox-Control


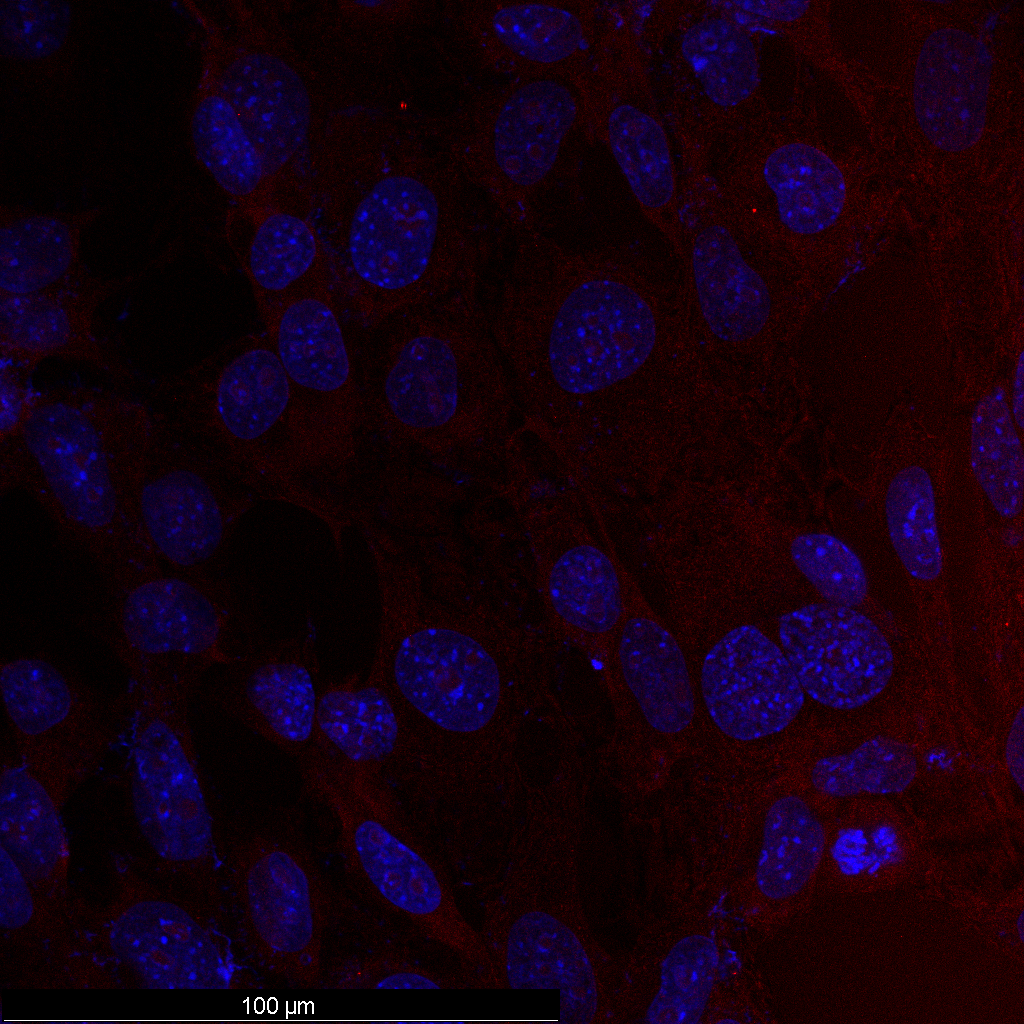


1. Mitosox-D-gal


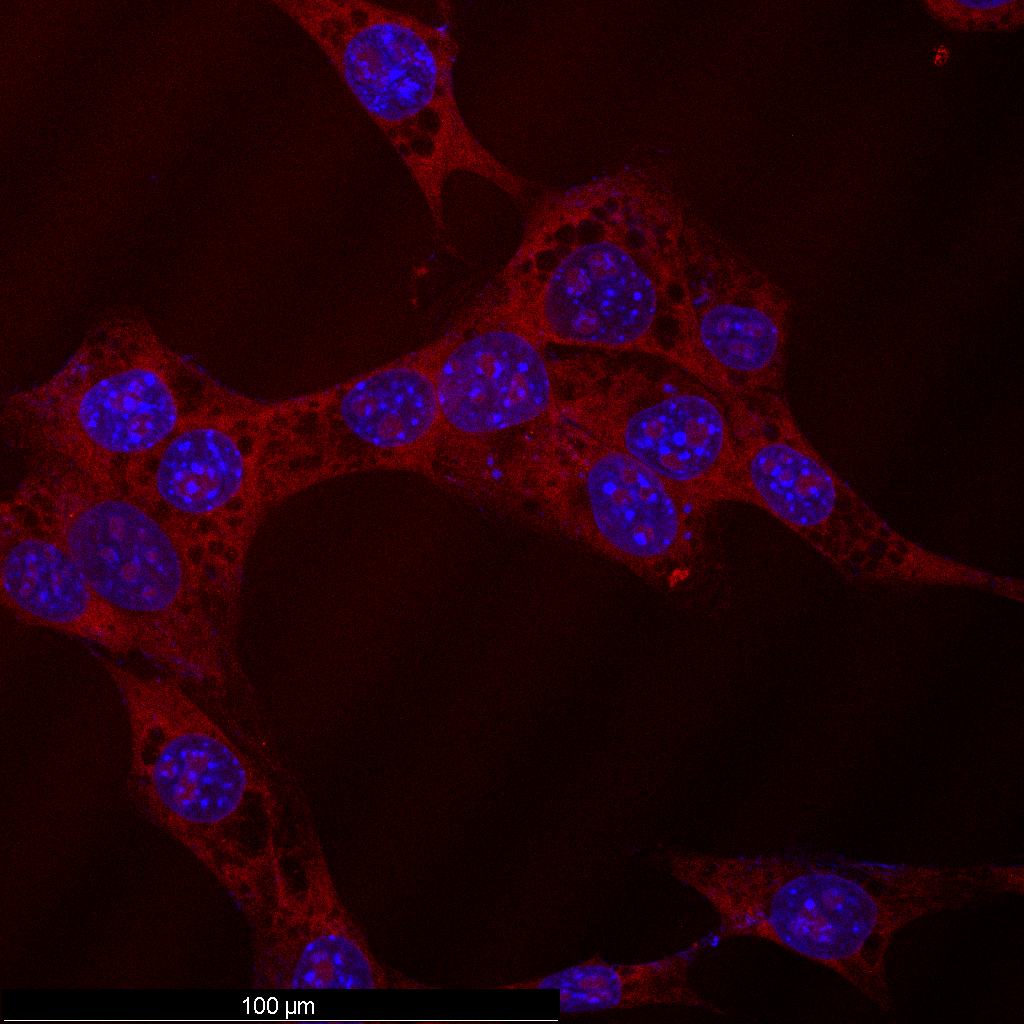


1. D-gal+5-HTP


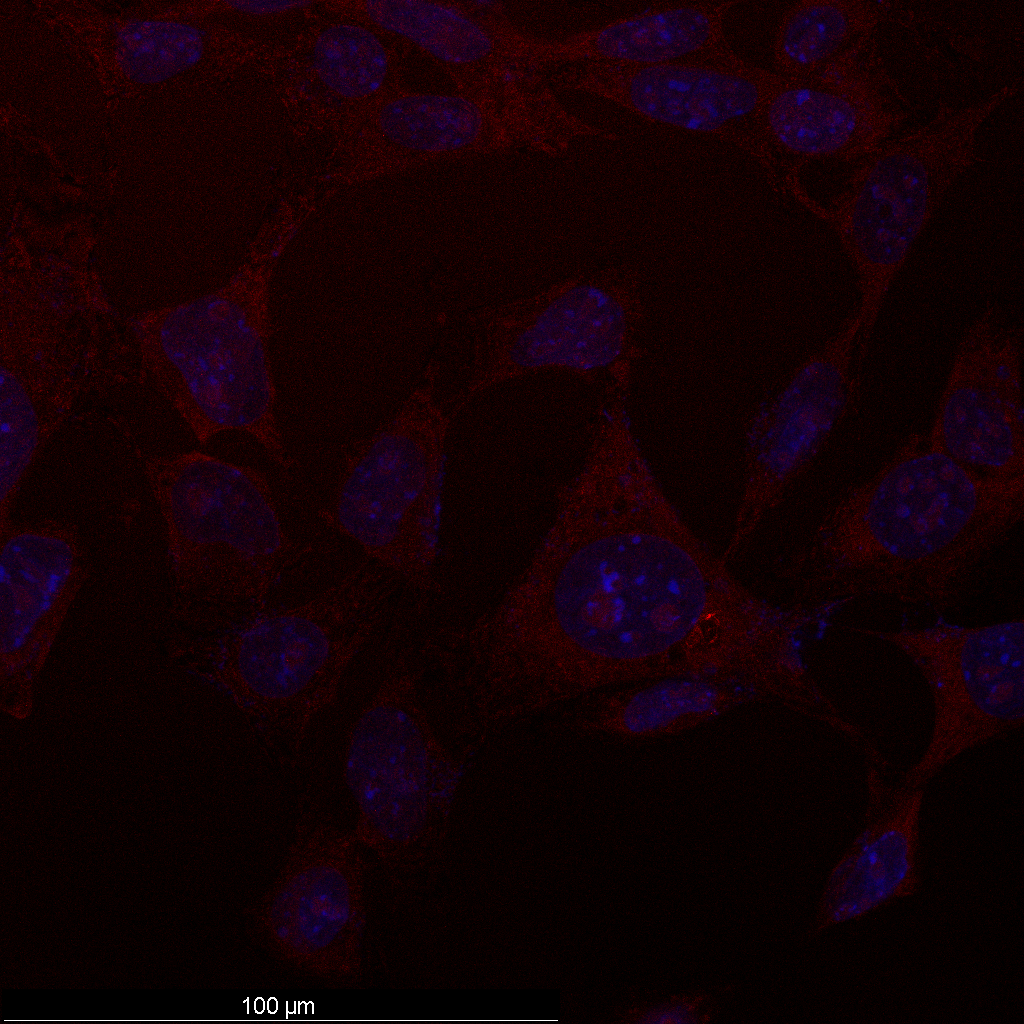


Figure 7G


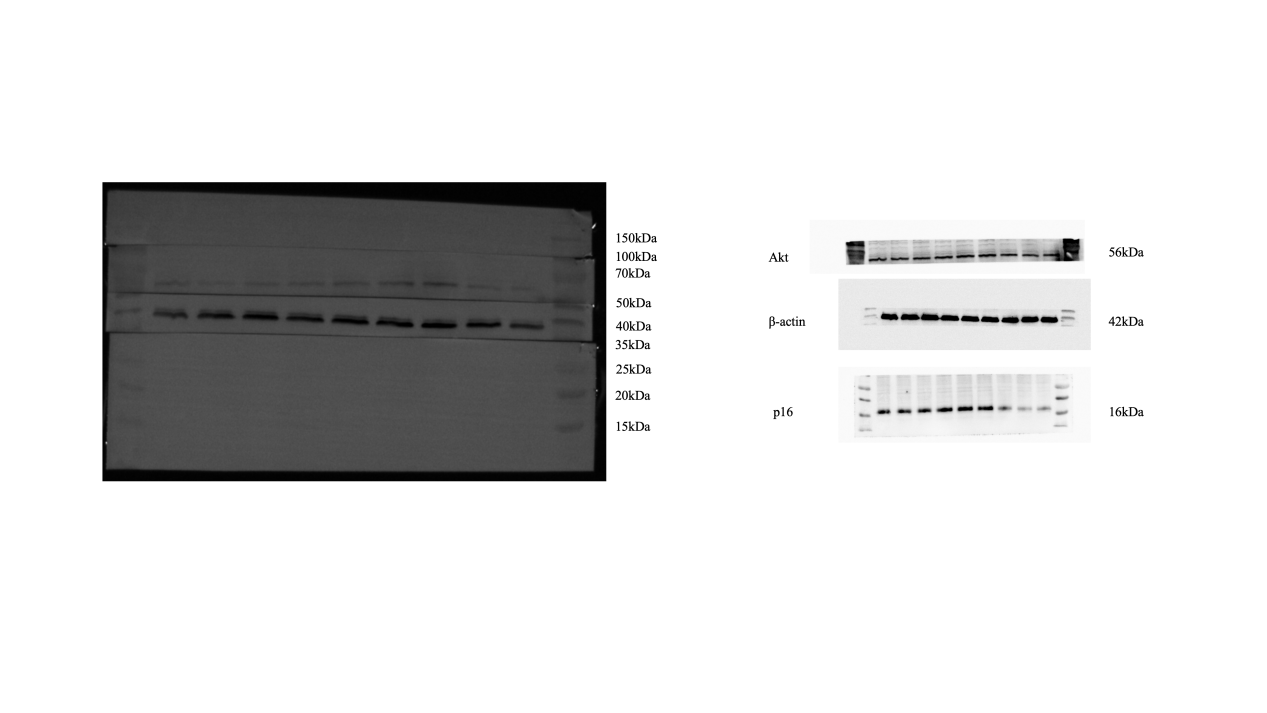


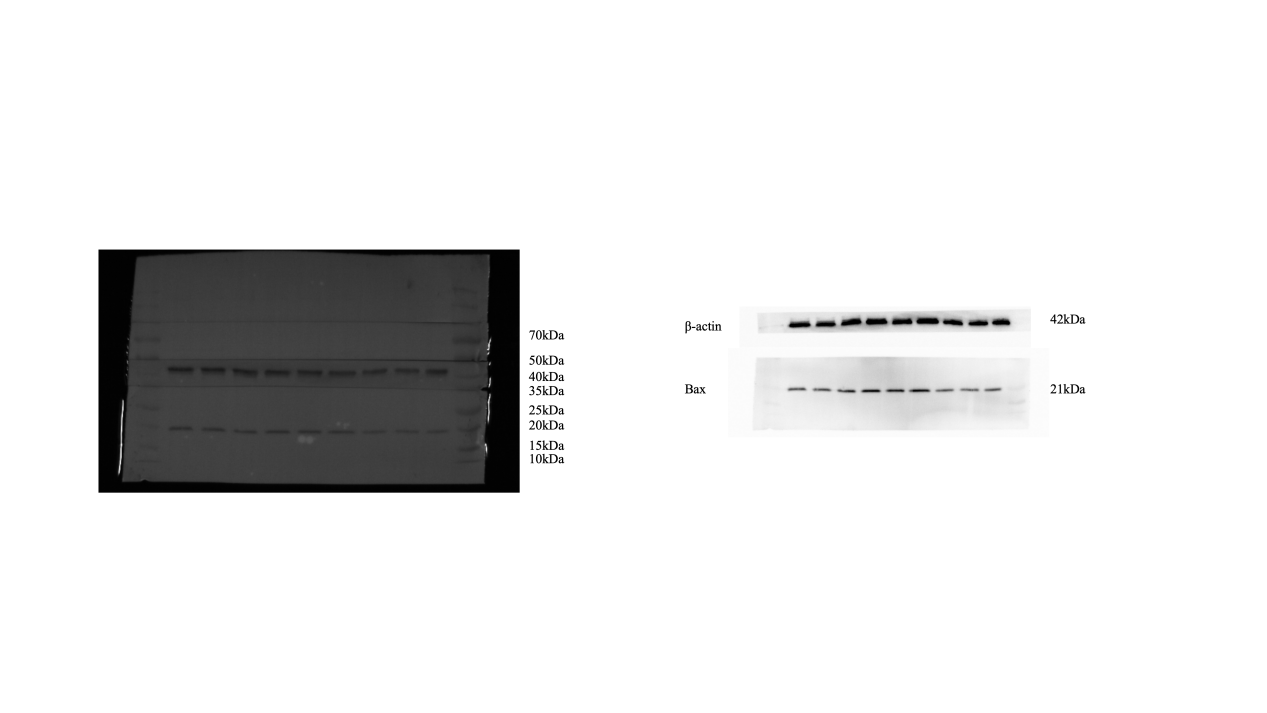


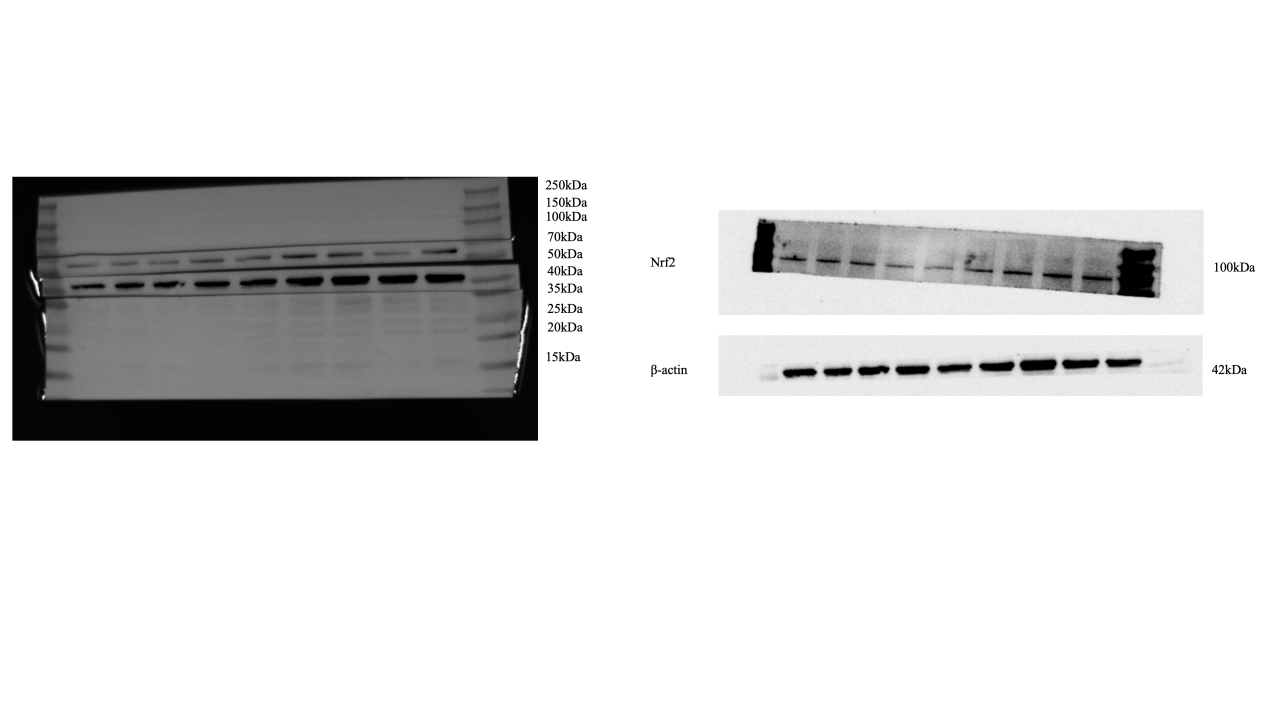


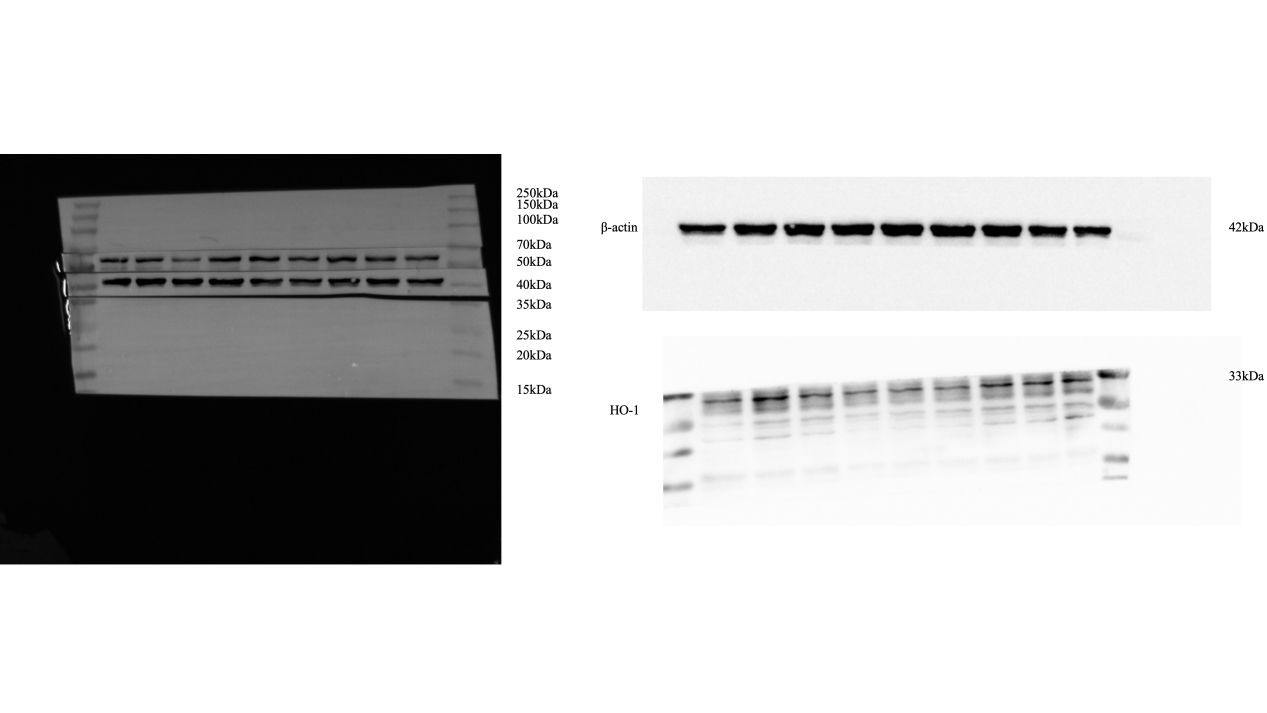


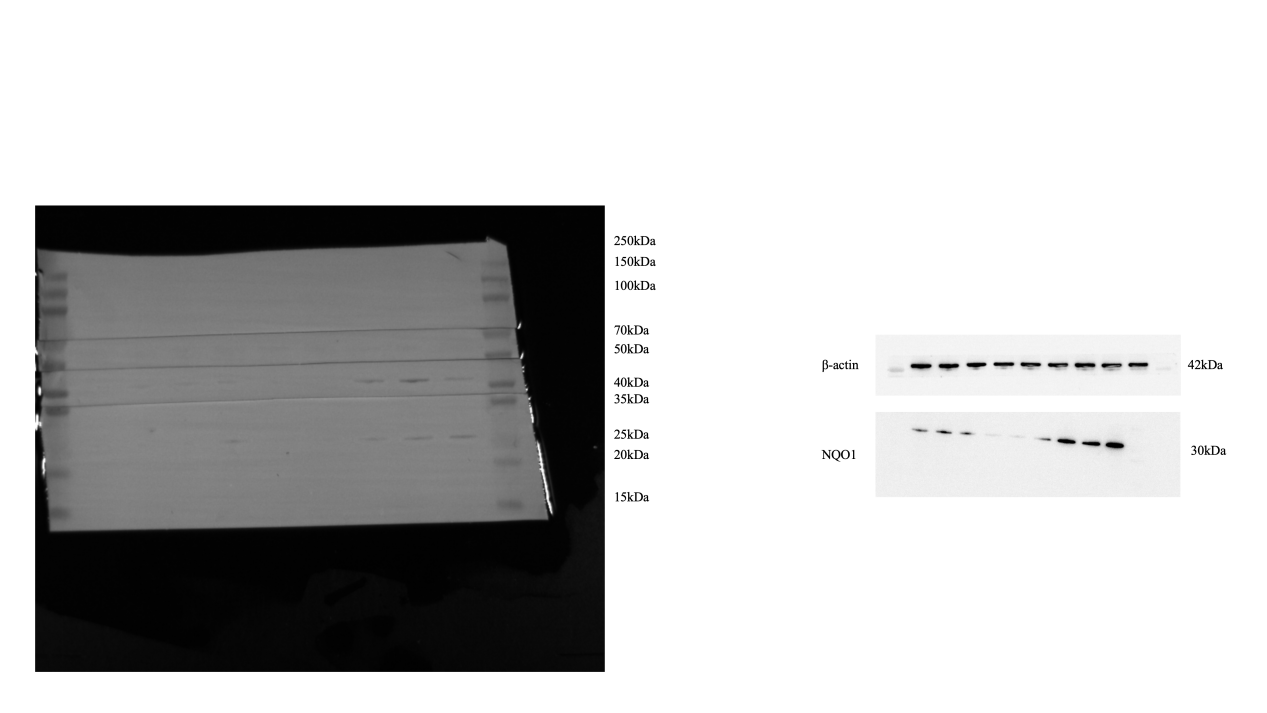


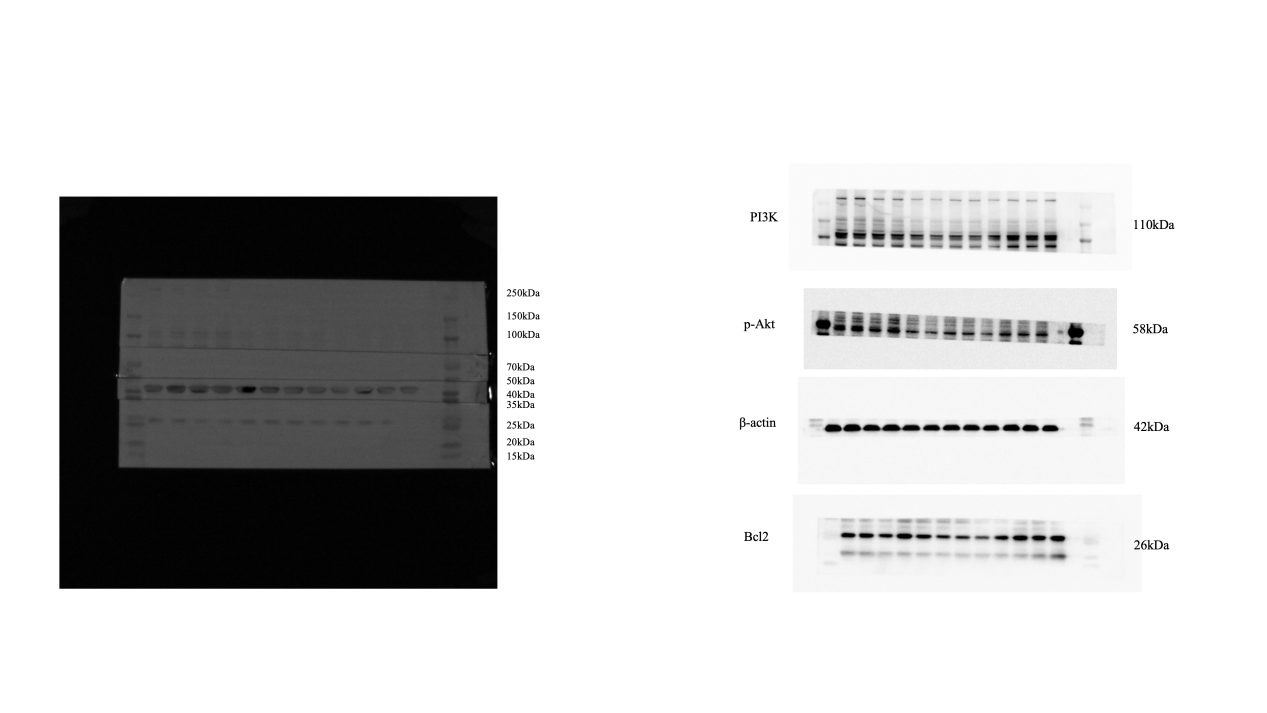

Supplement: Supplementary file 1 — Supporting File 1: advs73974‐sup‐0001‐SuppMat.docx [file ADVS-13-e14269-s002.docx]
